# Identification of suicide brain transcriptomic signatures using meta-analysis of multiple cohorts

**DOI:** 10.1038/s41398-026-03978-8

**Published:** 2026-03-31

**Authors:** Aleksandr V. Sokolov, Muataz S. Lafta, Jussi Jokinen, Helgi B. Schiöth

**Affiliations:** 1 Department of Surgical Sciences, Functional Pharmacology and Neuroscience, Uppsala University, Uppsala, Sweden; 2 Department of Clinical Neuroscience/Center for Psychiatric Research, Karolinska Institutet, Stockholm, Sweden; 3 Department of Clinical Sciences/Psychiatry, Umeå University, Umeå, Sweden; 4 Laboratory of Pharmaceutical Pharmacology, Latvian Institute of Organic Synthesis, Riga, Latvia

**Keywords:** Molecular neuroscience, Biomarkers

## Abstract

Suicide remains a critical global public health issue, accounting for nearly one million deaths annually and imposing profound societal and economic burdens. Despite its urgency, the lack of diagnostic and predictive biomarkers continues to hinder the development of effective prevention and treatment strategies. This study presents a comprehensive meta-analysis that integrates publicly available postmortem brain transcriptomic datasets and a domestic cohort, encompassing 16 cohorts. The transcriptomic data, sourced from the Gene Expression Omnibus repository, were generated using various techniques, including traditional RNA sequencing, microarray methods, and single-cell RNA sequencing. Differential expression analyses were performed across multiple brain regions, with meta-analyses stratified by cortical regions, the dorsolateral prefrontal cortex (DLPFC), and combined. We further analyzed whether covariates may affect the identified genes. Three meta-analytic approaches were employed, complemented by pathway and cell-set enrichment analyses. The unadjusted meta-analysis consistently identified several genes with altered expression, including upregulated P2RY12, CX3CR1, and GPR34, and downregulated SOX9 and PMP2, all at nominal significance. Additionally, multiple genes encoding long non-coding RNAs (lncRNAs) exhibited nominally altered expression in suicide, including RP5-837J1.4, AC159540.14, DNM1P47, AC004158.2, EEF1A1P30, and RP11-339B21.8. Several alternative strategies to run meta-analysis were performed and moderators were investigated. Cell-type-specific expression deconvolution and meta-analysis identified several genes overlapping with bulk expression meta-analysis, and genes were attributed to neuronal lineages. These findings highlight plausible molecular targets for future validation studies, suggesting the involvement of microglia (P2RY12 and CX3CR1), astrocytes (SOX9), immune responses (GPR34), myelin regulation (PMP2), and epigenetic modulation via lncRNAs. This research advances the understanding of the molecular architecture of suicide and provides a foundation for future studies focused on targeted prevention and therapeutic interventions.

## Introduction

Suicide is a major global public health issue and one of the top three causes of death among young people. The World Health Organization estimates that nearly one million people die by suicide each year worldwide, with the number of suicide attempts being several times higher than the number of suicide deaths [1, 2]. Numerous studies have highlighted the substantial economic burden of suicide, both in direct and indirect costs [3]. Suicide rates are higher in high-income countries compared to middle- and low-income countries, with men accounting for nearly three times as many suicides as women, particularly in high-income regions. Age stratification shows that suicide rates are highest among adults aged 70 and older in both genders [4]. Despite the imperative need to address this global health crisis, the current lack of diagnostic and predictive biomarkers significantly hinders suicide prevention, treatment, and the development of novel therapeutics [5–7].

The growing number of large-scale genome-wide association studies (GWASs) in recent years has significantly contributed to identifying gene variants linked to modest increases in suicide risk [8]. The latest GWAS meta-analysis, conducted by the International Suicide Genetics Consortium (ISGC), analyzed over 29,000 cases of suicide attempts or suicides from 18 cohorts worldwide [9]. However, the findings revealed an estimated heritability of approximately 6.8% for suicide attempts, emphasizing the role of genetic factors in this complex behavior. However, despite multiple research efforts, GWAS did not identify consistent suicide genetic markers [10], suggesting a potential role of other factors in suicide.

The use of postmortem brain studies has been pivotal in advancing our understanding of suicide and the impact of social stress on brain dysfunction [11]. For instance, reviews have reported molecular differences in the hippocampus of suicide completers and dysregulation of the hypothalamic–pituitary–adrenal (HPA) axis [12–15]. Other studies have reported suicide-specific molecular differences and gene associations [16], while some have identified structural differences in the brain across various types of suicidal behaviors [17].

**Fig. 1:**
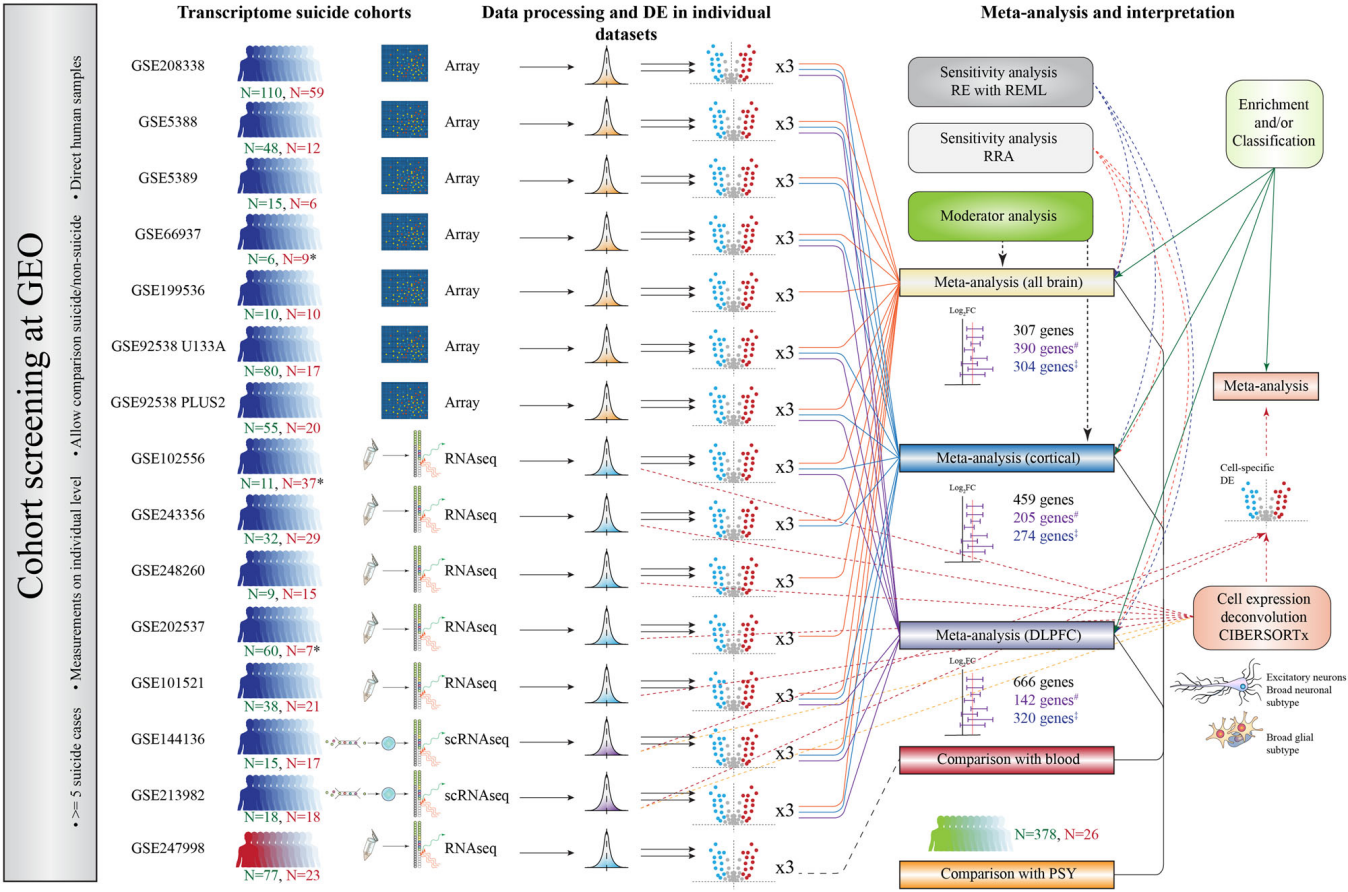
This figure shows the flowchart of the analyses in this work. First, data was collected from GEO using specified search terms (see Data S1) to obtain suicide-related transcriptome cohorts. Red number indicates the number of suicide cases, whereas the green number shows the number of non-suicide individuals. Asterisk indicates that the numbers for the cohorts GSE66937, GSE102556 correspond to prefrontal cortex samples and to Nac samples in GSE202537. Then, these cohorts passed to data preprocessing steps depending on their transcriptome measurement technology and then were subject to DE analyses. DE analyses were conducted both with and without covaries. Then, obtained DE results (with and without covariates) were meta-analyzed in either one, two or three meta-analyses depending on the tissue. Each colored arrow indicates corresponding meta-analysis. Hashtags indicate gene counts obtained from the analyses with covariates. Crossed dagger indicates results from analysis with surrogate variables. Obtained differentially expressed genes from meta-analysis were analyzed for overlaps, compared to blood cohorts GSE247998 and PSY cohort, and were subject to gene ontology overrepresentation analysis and classification with GPT-5. Meta-analyses were also tested for sensitivity and moderation. Data from RNAseq cohorts was deconvoluted with CIBERSORTx and meta-analyzed by selected cell types. Single-cell RNA seq data was used directly in cell-specific meta-analysis and to construct signature matrices. GEO, gene expression omnibus; Nac, Nucleus accumbens; DE, differential expression; RE, random effects model; REML, restricted maximum likelihood; RRA, robust rank aggregation.

Previous meta-analyses on gene expression alterations in suicide [18] have primarily focused on investigating specific components, like transcription factors, that regulate the molecular mechanisms of the suicidal brain. To our knowledge, the largest previous meta-analysis on molecular targets in suicide utilized only six cohorts [19]. In the current study, we conducted a more comprehensive, hypothesis-free quantitative assessment using publicly available transcriptome datasets related to completed suicide, sourced from the GEO repository [20], encompassing 14 post-mortem brain open-access cohorts. Our goal was to identify gene expression signatures consistently associated with suicide death by first performing individual analyses on each cohort, followed by a combined meta-analysis. We standardized the raw data across all cohorts using the same methodology and analysis pipeline, ensuring consistency before conducting the meta-analysis. The outline of this study is shown in Fig. 1.

## Materials and methods

Due to the extensive amount of the methods used, the main text contains primary aspects of the methods, whereas the extensive description is provided in the supplementary materials (Data S1). All of the associated analysis code is publicly available at https://github.com/AleksandrVSokolov/suicide_meta.

### Ethics declaration

The current work uses data from 15 public-access cohorts and one domestic cohort. The Psychiatric Health in Adolescent Study (PSY cohort) was performed in Uppsala, Sweden. The study was approved by the Regional Ethics Committee of Uppsala. All participants gave their written informed consent for participation. Data from 15 public-access cohorts were obtained from Gene Expression Omnibus (GEO) [20]. The use of data from GEO was approved by the Regional Ethics Committee of Uppsala. These studies were also approved by their corresponding national ethical review committees. Further information is available in the corresponding GEO records and/or in the initial publications.

### Cohort identification

The identification of suicide cohorts was performed on July 17, 2024, querying the GEO repository with the two following search terms:

1. (“suicide”[MeSH Terms] OR suicide[All Fields]) AND (“human”[Organism] AND (“Expression profiling by array”[Filter] OR “Expression profiling by genome tiling array”[Filter]))
2. (“suicide”[MeSH Terms] OR suicide[All Fields]) AND (“human”[Organism] AND “Expression profiling by high throughput sequencing”[Filter])

To be included in the analysis, GEO cohorts were required to pass all the following criteria:

1. A study must have confirmed suicide cases. Studies on psychiatric diagnoses, such as major depressive disorder, were not analyzed without clear identification of individuals who committed/attempted (live samples in blood) to commit suicide.
2. A dataset must have at least 5 suicide cases.
3. Expression signals or counts must be available at the resolution of the individual participants and individual transcripts.
4. Study design and study methods must allow comparison between suicide and non-suicide groups.
5. The study must have been conducted on human subjects and must involve human samples and not their derivatives (such as cell cultures).

The PSY cohort was included as a separate analysis (not a part of meta-analysis) since the research group had access to it. A detailed description of the differential proteomic profiling in the PSY and its demographic data is provided in the supplementary materials (Data S2). In total, we collected 15 public datasets from the following GEO repositories: GSE208338 [21, 22], GSE5388, GSE5389 [23], GSE66937 [24], GSE199536 [25], GSE92538 U133A, GSE92538 PLUS2 [26–29], GSE102556 [30], GSE243356 [31], GSE248260, GSE247998 [32], GSE202537 [33], GSE101521 [34], GSE144136, and GSE213982 [35, 36]. Demographic data for all identified GEO cohorts could be found in Data S3.

### Transcriptome array data preprocessing

Gene expression profiling was performed via transcriptome arrays in the following cohorts: GSE208338, GSE5388, GSE5389, GSE66937, GSE199536, GSE92538 (U133A), GSE92538 (U133 PLUS2). We used already available preprocessed data from GEO for GSE208338 as it was adjusted for batch effects that were not included in the phenotype data in GEO. In all other array-based cohorts, we prepared the data starting from raw .CEL files. Data preprocessing, filtering, and quality control procedures were performed in R (version 4.2.3). For details regarding data preprocessing steps, please refer to Data S1.

### Bulk RNAseq data preprocessing

We processed data from RNAseq reads deposited on the NCBI SRA portal. We used FastQC [37] and fastp [38] tools to perform quality control of the first few runs in every cohort to see if sequencing data had been preprocessed or contained sequencing adapters or other issues. Data preprocessing steps were performed sequentially using a custom Python script. Sequencing runs were obtained through the SRA toolkit and aligned with the *STAR* aligner [39]. Analysis was performed within the conda environment on a Linux machine (Ubuntu 22.04.2 LTS). Once counts were obtained, corresponding data and associated mapping and quantification statistics from STAR were imported into R. We inspected mapping percentages and count statistics to identify potentially problematic samples. One run in GSE102556 (SRR5961809) was excluded due to low mapping percentage. Three runs in GSE202537 (SRR19147540, SRR19147610, SRR19147547) were excluded due to very low read counts compared to the rest of the samples. Mapping of other runs was deemed as being of sufficient quality. The obtained counts were further used in differential expression analysis. A detailed description of bulk RNAseq data preprocessing methods is provided in the supplementary materials (Data S1).

### Single cell RNAseq data preprocessing

In the datasets GSE144136 and GSE213982, read data was generated from single-cell runs. As raw counts were available for every cell and gene and both datasets were preprocessed in a consistent manner [36], we decided to use these counts directly. The sparse matrix was imported in R via the readMM function from the *Matrix* package. Then the obtained matrix was imported into the *Seurat* R package [40]. We used cell annotation and meta-data available from the initial publication [36] and its GEO record. Obtained single cell data was pseudo-bulked in two ways: by individual and by both cell and individual. To take into account differences in cell compositions, we estimated cell proportions for every individual. Runs for the male participant 25 were excluded from the count matrix in the initial study due to failed quality control. Runs for the male participant 24 were split into two for unknown reasons, and we also decided to exclude them for better comparability between samples. For the same reason, runs for female participants 19 and 5 were excluded as they were run on a separate sequencing platform (BGI DNB-seq) from all other female samples. The obtained pseudo-bulk counts were further used in differential expression analysis.

### Differential expression analysis in the primary cohorts

Differential expression analysis in the individual cohorts was performed via the R package *limma* that uses the Empirical Bayes method to moderate expression variance during fitting of linear models [41]. RMA-normalized signal intensities were used for analysis with *limma* in array-based cohorts. If a cohort had more than one tissue, limma-based analysis was performed separately for every tissue. In RNAseq cohorts, raw transcript counts were first imported into the *DGEList* object from the edgeR package [42] and filtered using the *filterByExpr* function. This function uses transcript filtering strategy for the specified experiment design matrix, keeping only those transcripts that have at least minimal acceptable gene count per groups of participants. This filtering is extensively described in the original publication [43]. After filtering, we calculated normalization factors for the DGEList object. To enable limma-based analysis, the obtained counts in the DGEList object passed through voom transformation [44] implemented in *limma*. Then voom-transformed counts were used in the standard *limma* analysis pipeline. If an RNAseq cohort had counts from more than one tissue, count filtering, voom preprocessing, and limma-based analysis were conducted separately for every tissue. Differential expression analysis pipeline was performed using *limma lmFit* and *eBayes* with default parameters (after voom for RNAseq). *TopTable* was used to obtain differentially expressed transcripts and standard errors (SE) for log_2_ fold changes were estimated directly from the *limma fit* object.

As there are multiple possibilities to conduct statistical modeling [45], we performed differential expression analysis with *limma* in three strategies. The first strategy only considered suicide status (binary) as a predictor without adjustment for covariates. The second strategy included suicide status (binary) and phenotype covariates that were deemed as relevant for analysis by authors (see further rationale for the approach in Section 1.10). The relevance of covariates was determined if it had the possibility to affect gene expression and outcome, whether it has a sufficient number of levels (for categorical variables), as well as how many samples may be excluded due to missing values. We performed all analyses as “complete case”, and participants with missing information regarding included covariates were excluded. If a covariate showed full dependence on the suicide status or other predictors, it was omitted. The list of relevant covariates was different between the cohorts as different phenotype information was available for every study in the first place. If a study had no extra covariates for analysis, the results without covariates were used instead. The third strategy considered adjusting initial limma models with suicide status (binary) and estimated surrogate variables (SVs) using the R package *sva*. We adjusted for a maximum of five surrogate variables per dataset, considering the number of samples in cohorts. In GSE5389 and GSE66937 we estimated 0 required SVs, therefore such models were conducted as without covariates.

The adjusted model descriptions for every cohort as well as details on SV analysis are provided in the supplementary materials (Data S1).

### Probe and transcript mapping to gene symbols

To enable comparison between studies, we needed to ensure that genes are named consistently across all datasets. First, probes in the array cohorts were mapped to gene symbols using provided annotation files from the latest versions from the manufacturers or GEO. If the annotation files provided insufficient/inconvenient data to map probes to genes, we used a Python connection to BioMart - Ensembl https://pypi.org/project/biomart/ to perform mapping of probe IDs to Ensembl gene IDs and symbols. This strategy was used in GSE208338, GSE66937, and GSE199536. Mapping of RNAseq reads to Ensembl gene IDs was performed by the *STAR* aligner directly. To ensure consistent naming of genes, we further harmonized names against an identical reference dataset with approved gene symbols and synonyms.

### Meta-analysis of differential expression changes

We performed a meta-analysis for differential expression changes for all included genes if they passed both of the following selection criteria:

1. A gene was analyzed in at least five brain datasets. Estimates obtained from blood were not formally meta-analyzed but were used as a side comparison.
2. A gene was considered analyzed in the study if it had at least one probe that was uniquely annotated to its symbol. All probes that were annotated/aligned to several genes were discarded as ambiguous.

In some of the datasets several probes analyzed the same gene. In this case, we generated an aggregated differential expression measure for such genes. The effect size for such a gene was calculated as an arithmetic mean of all individual log_2_FC for the gene in the study. Standard error (SE) for an aggregated measure was formulated as the maximal SE among all log_2_FC SEs observed for the gene in the study. Such a strategy provides the largest confidence interval (the lowest certainty) and is a conservative approach to represent gene expression measured by several probes.

$$\begin{array}{l}{Aggregated}\,{Lo}{g}_{2}{FC}=\frac{{\sum }_{i=1}^{N}{Lo}{g}_{2}F{C}_{i}}{N}\\ {Aggregated}\,{SE}=\max \left(S\right),{where}\,S=\{S{E}_{1},S{E}_{2},...,S{E}_{n}\}\end{array}$$

Here N denotes the number of probes corresponding to the same gene in a single study. The letter S denotes a set of standard errors for log_2_FC estimates for the same gene, and SE denotes a standard error.

Since many potential strategies and factors could be considered when meta-analyzing individual studies, we focused on the three most straightforward directions that were deemed reasonable based on the authors’ subjective judgment:

1. Meta-analysis of brain expression in all brain datasets (only one tissue per cohort). For GSE202537, we selected Nucleus accumbens (Nac) to represent the cohort as it had the largest number of available probes. In GSE102556 and GSE66937, we selected prefrontal cortex expression as it was the most common tissue in all cohorts. In total, 14 datasets were meta-analyzed.
2. Meta-analysis of brain expression in all cortical datasets (only one tissue per cohort). We included the following tissues: dorsolateral prefrontal cortex, orbitofrontal cortex, and temporal cortex. In the cortical meta-analysis, 11 datasets were meta-analyzed.
3. Meta-analysis of brain expression specifically in the dorsolateral prefrontal cortex. Here we included only 9 datasets corresponding to the prefrontal cortex (GSE66937 unspecified) and dorsolateral prefrontal cortex. Cohort GSE5389 was not included as it was specifically collected using orbitofrontal cortex samples. GSE66937 was included as the authors assume primarily dorsolateral origin of these samples, even though it was not clearly indicated in the initial study.

Each of these specified meta-analyses was performed in three versions: *limma* estimates obtained without adjustment for covariates, with adjustment for covariates, or with adjustment for surrogate variables. Thus, nine primary meta-analyses were conducted in total. In the meta-analysis without covariates, the effects from suicide-related factors (such as depression/schizophrenia diagnosis) and suicide status are not separated and are considered as a part of the same causal pathway. This analysis should capture genes that are associated both with suicide and with suicide-related psychiatric diagnoses and phenotypes. In the adjusted analyses, the effects of co-morbidities are controlled for (depending on the dataset) and primarily suicide-specific changes, in theory, should be identified. Surrogate-variable adjustment, on the other hand, is expected to adjust unknown variation in the datasets in a standardized manner.

The meta-analysis model was based on the random-effects [46] approach implemented in the R package *metafor* with the function *rma.uni* [47]. As an input, we used *limma*-estimated log_2_FC (effect size) and its associated SE (vectors of the same length, n > =5). SE was squared and used as a sampling variance input (see *metafor* documentation). Weights for individual studies were calculated using the inverse-variance methods (*metafor* default). To enable model convergence for all genes, the amount of heterogeneity was set to the Sidik-Jonkman estimator (also called model error variance estimator) [48, 49]. This estimator was selected due to simplicity (closed-form solution) and as we expected to observe high heterogeneity across studies, and this estimator was shown to provide the most conservative estimate for heterogeneity with high positive bias for small heterogeneity and small bias if true heterogeneity is high [49, 50]. We considered the nominal significance of genes after meta-analysis. We also report FDR-corrected *p*-values calculated via the R function *p.adjust* (method = “fdr”) for information purposes, though they were not further used. To select the most important genes, we focused on transcripts with absolute log_2_FC ≥ 0.2.

### Sensitivity analysis and moderation analysis

We performed an alternative calculation for meta-analysis, using the restricted maximum likelihood estimator (REML) with parameters control=list(stepadj=0.5, maxiter=10000) to enable convergence for all transcripts and applied Hartung-Knapp-Sidik-Jonkman correction by setting test = “knha” (as suggested by the *metafor* documentation).

Additionally, we performed an alternative calculation for meta-analysis using robust rank aggregation (RRA) [51]. This strategy is based on relative ranking of genes within ordered lists. To consider the directional nature of log_2_FC, RRA was performed separately for upregulated and downregulated lists from every initial cohort-level differential expression datasets. Further details on RRA are available in Data S1 (Section 1.11).

Further, we investigated how cohort-level moderators, such as platform (array or RNAseq), mean PMI, percentage of male participants, and percentages of depression, bipolar disorder, or schizophrenia participants may be related to observed effect sizes. This analysis was performed with a mixed-effects model implemented within the *rma.uni* function where moderators could be specified as parameters. Due to extensive study number requirements for such analysis [52], we were able to investigate moderators only for genes that were present in at least 10 datasets. Further details regarding moderator selection and analysis are available in Data S1 (Section 1.11).

### Cell deconvolution meta-analysis

Due to its length, we provided a detailed description of cell deconvolution meta-analysis in Data S1 (Section 1.12). Briefly, we used two signature matrices (large sampled signature matrix simplified (LSSMS) and sampled signature matrix) obtained from single-cell profiles of GSE144136 and GSE213982 and CIBERSORTx [53] to deconvolute collected bulk RNAseq datasets. Primary RNAseq datasets were analyzed directly. First, gene lists were filtered, and then we performed DE analysis in every deconvoluted dataset for broad “neuronal” and “glial” profiles, as well as excitatory neurons (“ExN”). Then, obtained DE genes were further filtered to include only hits that are already nominally significant in GSE144136 or GSE213982 and then were meta-analysed using random-effects model in *metafor* similar to primary pipeline described in Section 2.8.

### Gene enrichment analysis and classification

Gene set enrichment analysis was applied for all genes significant in the meta-analysis (default approach). As a universe, we set all genes that were available for analysis. Mapping of genes to ENSEMBL gene IDs was performed using NCBI gene dataset: https://ftp.ncbi.nih.gov/gene/DATA/GENE_INFO/Mammalia/. Enrichment analysis was performed for biological processes using the *clusterProfiler* R package [54]. Additional gene classification was performed for significant genes with absolute log_2_FC ≥ 0.2. We used the GPT-5 model and manual curation to perform classification. More details are provided in the supplementary materials (Data S1).

## Results

### Analysis strategy and rationale

The main idea behind the following analysis was to investigate transcriptomic suicide markers and their stability across different cohorts and populations. Investigation of suicide transcriptome poses significant multiple comparison challenges due to very small sample sizes in the individual datasets (due to the nature of post-mortem brain samples) together with many potential transcript candidates. Since large-scale post-mortem brain cohorts are not likely to ever be collected, our priority was to consider effect sizes as the main evidence for gene importance with nominal significance in the meta-analysis. We did not restrict the list of investigated genes to have the highest possible coverage and to avoid positive result bias [55]. Such a strategy could be considered relevant due to the exploratory nature of the present work [56–62]. We only meta-analysed genes whose transcripts passed quality control in at least five cohorts as we did not expect that meta-estimates from a lesser number of studies would be of sufficient quality [63].

The inherent difficulty of every statistical analysis is the consideration of relevant covariates that could be included in the model as well as multiple possibilities that the same analysis could be conducted differently resulting in the multiple comparison problem [45, 64, 65]. The comparison of studies becomes even more complicated as the set of covariates available differs between studies. Thus, we present this work as a multiverse analysis [45] where we considered several important factors that could affect the outcome, including tissue specificity, the use of covariates or surrogate variables to obtain initial confidence intervals, as well as the use of different heterogeneity estimators to estimate heterogeneity between the studies. We also considered a completely alternative strategy based on gene ranking (robust rank aggregation) as well as the use of study-level moderators for meta-analyses. (see results and Data S1).

### Generation of datasets

This study includes a total of 16 cohorts: 15 open-access cohorts (14 transcriptomics and one blood-based) and one domestic cohort (PSY) (Data S2 and S3). The PSY cohort consists of 404 participants, including 26 suicide cases and 378 controls, with equal sex distribution across both case/control groups (77% female and 23% male). The open-access cohorts vary in size, ranging from the largest cohort, GSE208338 with 169 participants, to smaller cohorts like GSE5389 with only 21 participants. These cohorts analyze differences between control and suicide groups by focusing on various brain regions, primarily the dorsolateral prefrontal cortex (DLPFC), as well as peripheral blood. The transcriptome data were derived from a variety of techniques across multiple datasets (Fig. 1). RNA sequencing (RNAseq) was the primary method used in several datasets, including GSE101521, GSE102556, GSE202537, GSE243356, and GSE248260. Single-cell RNA sequencing (scRNAseq) was applied in GSE144136 and GSE213982, while microarray technology was used for transcriptomic profiling in GSE199536, GSE208338, GSE5388, GSE5389, GSE66937, GSE92538_U133_PLUS2, and GSE92538_U133A.

Across the datasets, suicide groups include participants with major depressive disorder (MDD), bipolar disorder (BPD), and schizophrenia (SCZ), with differences in participant numbers and the specific brain regions assessed. DLPFC-based cohorts include GSE92538 PLUS2, GSE92538 U133A, GSE208338, GSE101521, GSE5388, GSE144136, and GSE213982. These cohorts vary in participant numbers, with distributions in the control and suicide groups as follows: GSE92538 PLUS2 includes 75 participants, GSE92538 U133A has 97, GSE208338 has 169, GSE101521 has 59, GSE5388 has 60, GSE144136 has 32, and GSE213982 has 36. The suicide participants in these cohorts primarily have diagnoses of MDD, BPD, and SCZ.

Several other brain regions are also examined in other cohorts. The temporal cortex, specifically BA20 and BA36, is analyzed in GSE243356, which includes 61 participants, 29 of whom are in the suicide group. GSE102556 focuses on multiple brain areas, including the orbitofrontal cortex (OFC, BA11), cingulate gyrus (Cg25), anterior insula (aINS), nucleus accumbens (Nac), and subiculum (Sub), with participant numbers ranging from 28 to 48, and primarily involving MDD suicide cases. GSE66937 provides data from the amygdala, hippocampus, thalamus, and prefrontal cortex with smaller samples, ranging from 14 to 15 participants. GSE202537 includes data from the caudate, putamen, and nucleus accumbens (Nac) and has 67-70 participants, with suicide cases diagnosed with BPD and SCZ.

Finally, one cohort examines peripheral blood. GSE247998 includes 100 participants, with 23 individuals in the suicide group, predominantly diagnosed with MDD.

### Differential expression analysis in the individual cohorts

In the analyses without the inclusion of covariates, a total of 1,519,004 models were tested, yielding 99,496 nominally significant associations (Data S5). The average absolute effect size (log_2_FC) across all transcripts was 0.088, while the average absolute effect size for nominally significant associations (p < 0.05) was significantly more pronounced at 0.265. Effect sizes ranged between individual datasets, with the largest values being observed in data obtained from scRNAseq. Some genes achieved FDR-adjusted significance (p < 0.05) in several cohorts: GSE102556 (4 genes in the subiculum), GSE144136 (11 genes in the dorsolateral prefrontal cortex, BA9), GSE202537 (8 genes in the nucleus accumbens), GSE213982 (3 genes in the dorsolateral prefrontal cortex, BA9), and GSE92538_U133_PLUS2 (1 gene in the dorsolateral prefrontal cortex, BA9).

When covariates were included in the models (Data S6), the total number of associations tested increased to 1,547,825 (due to increased number of probes that passed filtering in RNAseq), with 78,392 associations reaching nominal significance. Surprisingly, the overall average absolute effect size was increased both for all associations at 0.14 and for nominally significant associations at 0.41. The largest effect sizes in this setting were observed in the cohort GSE102556. Interestingly, no genes achieved FDR-adjusted significance (p < 0.05) when covariates were included. This indicates that the inclusion of covariates reduced the number of nominally significant associations despite the increased magnitude of effect sizes.

Inclusion of surrogate variables in the initial DE models did not change the number of tested associations as these covariates were available for all samples. Contrary to dataset-derived covariates, surrogate variables decreased effect sizes for all associations (0.076) and nominally significant associations (0.23). In total, 89,207 associations reached nominal significance, and several genes also passed FDR-adjusted significance in several cohorts, with the largest number N = 11 in GSE92538 (U133 PLUS2 subset) (Data S7).

### Random effects meta-analyses

**Fig. 2:**
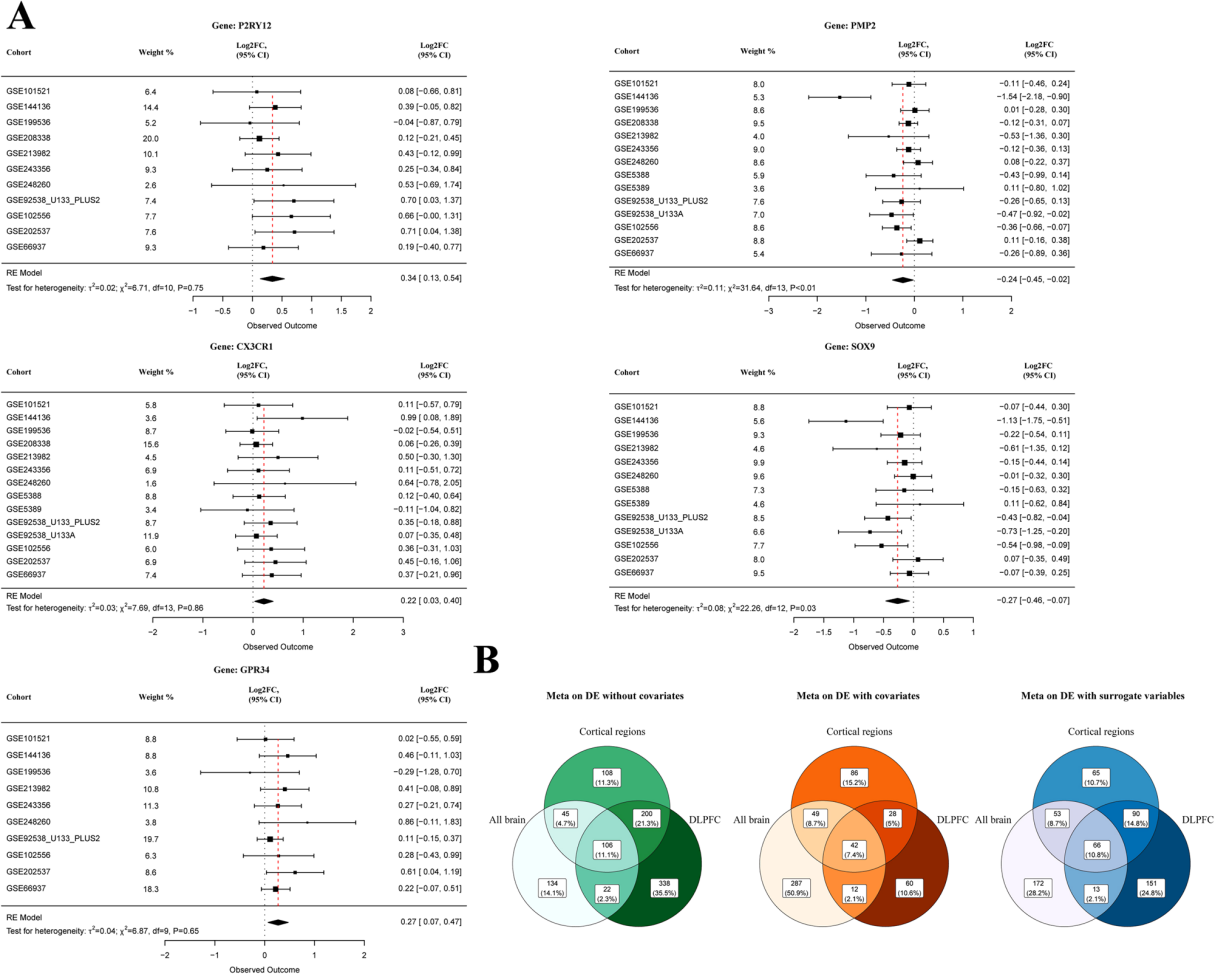
Main meta-analysis results. **A** This figure shows five forest plots for genes with highest log_2_FC in the meta-analyses without covariates that overlapped between tissues and are supported by literature to be related to suicide and psychiatric disorders. Forest plots were created in the R package *metafor*. **B** This figure contains Venn diagrams indicating the number of significant genes (nominally) obtained in each meta-analysis with covariates, without covariates, and using surrogate variables. Each number indicates the number of genes.

**Fig. 3:**
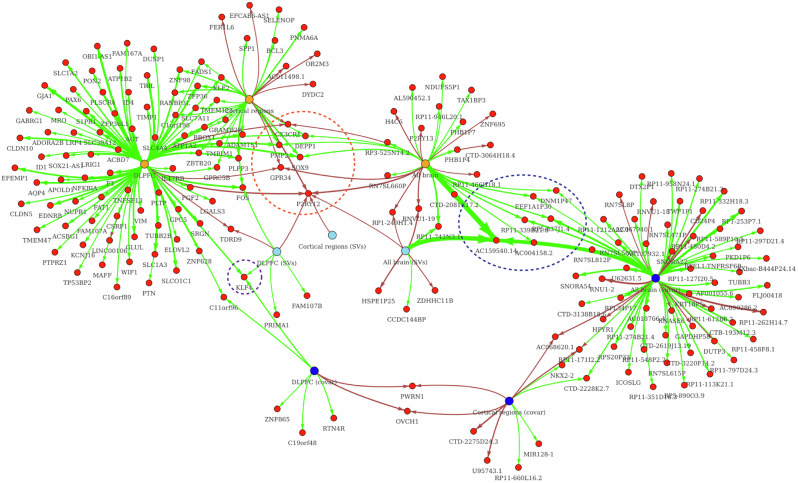
This figure shows network visualization for nominally significant results obtained in all meta-analyses. Only the genes with meta-estimated absolute log_2_FC ≥ 0.2 Yellow nodes indicate meta-analyses without covariates. Blued nodes indicate meta-analyses with covariates. Light-blue nodes indicate analysis with surrogate variables. Red nodes show genes. Arrow edges indicate nominal significance for a gene in the corresponding analysis. Edge colors correspond to direction where red indicates upregulation in cases and green indicates downregulation. Edge thickness is proportional to the absolute values of log_2_FC. Orange dashed area indicates genes that were significant in all three meta-analyses without covariates. Blue dashed area indicates genes that were significant in meta-analysis both with and without covariates in all brain tissues. Violet dashed area shows genes that were nominally significant in DLPFC regardless of the use of covariates, surrogate variables, or no adjustment.

The differential expression analysis results from each cohort were meta-analyzed with a random-effects (RE) model to assess gene expression changes across all included genes. A total of nine meta-analyses were conducted, segmented by specific brain regions (all brain regions, cortical regions, and dorsolateral prefrontal cortex (DLPFC)) and by analyses with and without covariate adjustment and the use of surrogate variables (Figs. 2 and 3; Data S8–S11).

As expected, none of the probes reached significance after the FDR correction in any of the analyses conducted. Without covariate adjustment, the meta-analysis identified 152 upregulated genes and 155 downregulated. In cortical regions, 261 hits were upregulated and 198 hits were downregulated. In the DLPFC, 331 genes were upregulated and 335 downregulated. After adjusting for covariates, these numbers changed to 183 upregulated genes and 207 downregulated in all brain analysis, 132 upregulated and 73 downregulated in cortical sub-analysis, and 92 upregulated and 50 downregulated genes in DLPFC analysis. The amount of heterogeneity (**I**^**2**^) was different depending on the studied transcript and analysis performed. However, it was generally estimated as high and was **I**^**2**^min = 6%, **I**^**2**^max = 98.6%, **I**^**2**^median = 49.2% in all brain tissues and had comparable values in tissue subsets. Heterogeneity for nominally significant genes was lower as expected, reaching **I**^**2**^median of 32.9% in all brain tissues, 34.4% in cortical tissues, and 35.3% for DLPFC. Interestingly, inclusion of covariates in meta-analyses decreased **I**^**2**^median by around 10% in every of the performed meta-analysis per tissue, with **I**^**2**^median reaching 39.9% (all brain), 37.9% (cortical), and 35.9% (DLPFC) for all analyzed probes. Surrogate variables also decreased heterogeneity in all tissues by around 9-12% (see Data S12).

In the unadjusted analyses, overlap across brain regions included 45 proteins (4.7%) between all brain and cortical regions, 22 proteins (2.3%) between all brain and DLPFC, 200 proteins (21%) between cortical regions and DLPFC, and 106 proteins (11.1%) overlapping across all three regions. In the analyses adjusted for covariates, the overlap included 49 proteins (8.7%), 12 proteins (2.1%), 28 proteins (5%), and 42 proteins (7.4%) for the same regions. None of these findings reached significance after correction for multiple testing.

In the unadjusted analyses, the top three upregulated genes were P2RY12, RNVU1-19, and GPR34 in all brain regions; P2RY12, AC011498.1, and OR2M3 in cortical regions; and P2RY12, CX3CR1, and TDRD9 in the DLPFC. The downregulated genes included RP11-742N3.1, RP11-339B21.8, and AC159540.14 in all brain regions; ZFP36, PMP2, and SOX9 in cortical regions; and TRIL, OBI1-AS1, and GJA1 in the DLPFC. After adjusting for covariates, the most upregulated genes were RP11-262H14.7, RNU1-2, and CD24P4 in all brain regions; CTD-2275D24.3, U95743.1, and RP11-171I2.2 in cortical regions; and OVCH1, PWRN1, and LRRC3B in the DLPFC. The top three downregulated genes post-adjustment were RP5-837J1.4, RP1-253P7.1, and AC159540.14 in all brain regions; MIR128-1, RP11-660L16.2, and CTD-2228K2.7 in cortical regions; and RTN4R, C11orf96, and KLF4 in the DLPFC. We observe that 57 nominally significant genes overlapped between the analyses without covariates and with covariates in all brain tissues. Among these, RN7SL8P, LRRC9, GPR160, GRB14, RP11-91G21.1, and GCNA had average log_2_FC ≥ 0.1, and 14 genes had log_2_FC ≤ -0.1. We computed similar statistics for other tissue sub-analyses, as well as comparison with the use of surrogate variables (Data S13).

Surrogate variable adjustment of initial DEs and subsequent meta-analysis generally identified different sets of nominally significant genes compared to no use of covariates and phenotype covariates. For example, if we consider all tissues, 30 (3.6%) genes were overlapping in all three strategies, 93 (11.2%) genes overlapped between surrogate variables and no covariates, whereas 6.6% of genes overlapped between surrogate variable adjustment and phenotype covariates (Fig. S1). Results with log_2_FC  ≥ 0.2 obtained in all meta-analyses are summarized in the network visualization in Fig. 3. *P2RY12* overlaps in six analysis strategies with matching association directions (upregulation in suicide cases), *AC159540.14, CX3CR1, DEPP1, FOS, GPR34, KLF4, PMP2*, and *SOX9* overlap in three strategies, respectively. *AC159540.14* demonstrated the largest absolute effect size (log_2_FC ~ -1.2), however, it was only analyzed in five initial datasets. Interestingly, KLF4 is the only gene that overlaps in DLPFC regardless of whether covariates, surrogate variables, or no adjustment were used.

**Fig. 4:**
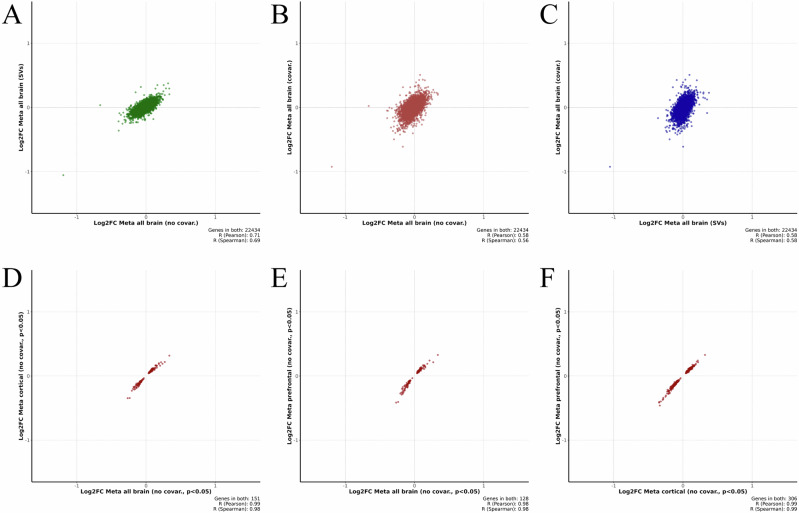
This figure shows pairwise correlations between estimated log_2_FC across different meta-analysis strategies with respect to not using covariates, using phenotype covariates, and using surrogate variables as covariates. These correlation plots were calculated on all overlapping genes without thresholding by log_2_FC or *p*-value (letters **A-C**). Letters **D-F** show correlations of meta-estimated log_2_FC with respect to subsetting analysis to specific tissues (all brain, cortical regions, or dorsolateral prefrontal cortex) for nominally-significant genes in the meta-analysis on DEs without covariates. DEs, differential expressions.

Meta-estimated effect sizes (log_2_FC) that were estimated on all analyzed overlapping genes (non-thresholded by log2FC and p-value) showed strong correlation between analyses without covariates and using surrogate variables (Rp ~ 0.71, Fig. 4A). However, the latter analysis with SV-adjusted initial DE tables shows lower estimated effects for all tissue subsets. Covariate-adjusted meta-analysis demonstrated more moderate correlations (Rp ~ 0.58, Fig. 4B) with analyses where covariates were not used as well as analysis using surrogate variable-adjusted DEs (Rp ~ 0.58, Fig. 4C). Consistently with correlations, approximately 77% of genes matched in meta-estimated log_2_FC direction, when no-use of covariates is compared with SVs in all brain tissue subset. A similar comparison of SVs with the use of phenotype covariates or phenotype covariates with no-use covariates, results in 71.7% and 70.6% overlap, respectively. This indicates a slightly higher similarity of SV-based approach to no-use of covariates than phenotype-based covariates (Data S13). However, it is less pronounced when compared to correlations and analyses generally overlap in terms of association directions. Restricting analysis to specific tissues had minor impacts on estimated effect size, potentially due to overrepresentation of cortical datasets. For instance, in the meta-analyses without covariates, all tissues subset, cortical subset, and prefrontal cortex subsets all demonstrated very strong correlations (Rp ~ 0.98, Fig. 4D–F) for nominally significant genes. In all genes (non-thresholded by log2FC and *p*-value), however, correlation was slightly lower (Rp ≥ 0.85, Fig. S2A-C.).

### Sensitivity and moderator analyses

Conducting meta-analyses involves many decisions that may significantly impact downstream steps. Therefore, we sought to perform potential alternative analyses and compare their outcomes with the main random effects model approach. We first conducted an alternative calculation for the RE meta-analysis using the restricted maximum likelihood estimator (REML) (Data S14). The results from this approach, incorporating the REML estimator for heterogeneity and the Hartung-Knapp-Sidik-Jonkman correction, showed substantially increased numbers of significant genes for all meta-analyses. For instance, 862 differentially expressed genes were detected in the meta-analysis with all brain regions without covariates, which indicates more than a 180% increase. These changes are potentially due to significantly decreased heterogeneity estimates from REML compared to the SJ estimator. For instance, **I**^**2**^median reached 12.7% in the analysis with all brain tissues without covariates, 12.9% in the cortical regions without covariates, and 14.5% in DLPFC, indicating an approximately 35% decrease in **I**^**2**^median on average. These estimates are likely substantially underestimated due to the diverse nature of RNA quantification methods and analyzed populations. Thus, for our further analyses and interpretation, we focused on more conservative estimates from SJ.

We also performed an alternative identification of suicide-related genes via robust rank aggregation (RRA) meta-analysis. This method identifies significant genes based on the relative positions of these genes in the set of ranked lists and how these positions are different from random lists [51]. As expected, this method produced relatively different results compared to RE models in all subsets of analysis (including covariate and SV usage, as well as different tissues, Data S15). Interestingly, this approach identified more nominally significant genes than RE in all subsets. For example, in all brain tissues without covariates, RRA captured 3016 genes compared to 307 in random effects. However, no genes were identified at FDR < 0.05 in all analysis strategies and tissue subsets. Spearman correlations of *p*-values were low (absolute r < 0.2) between RE models and corresponding RRA models. Some genes overlapped between RE and RRA in several analyses (Data S15 and Fig. S3). For example, in all brain subsets without covariates, 40 genes were nominally significant in both methods. In the network visualization Fig. S3, where we pruned associations from RE by significance in RRA (p_nominal_<0.05) and absolute log_2_FC ≥ 0.1, we could observe that transcripts, such as *GRB14*, *C1orf198*, *LY86-AS1*, and *PMP2* are supported by at least four analyses.

Lastly, we explored how cohort moderators may explain variation in observed effects (Data S16). Due to the small number of cohorts, coefficients in meta-regressions generally failed to reach statistical significance in most genes. We further explored how effects of nominally significant genes from primary RE meta-analysis were related to moderators. Average R2 (explained heterogeneity) was between ~19% and 32% depending on tissue subset and the use of covariates in the primary DE analyses. The number of genes where at least one moderator was statistically significant was the highest in meta-analysis from DE without covariates and the lowest from DE with covariates. Interestingly, beta coefficients for percentages of psychiatric disorders moderators demonstrated strong correlations in the cortical subset of analysis (Fig. S4), potentially suggesting a shared biological role of the genes in these disorders in the context of suicide. Among the most promising genes obtained in the primary RE meta-analysis (including *P2RY12*, *AC159540.14*, *CX3CR1*, *DEPP1*, *GPR34*, *KLF4*, *PMP2*, and *SOX9*, as well as *GRB14*, *C1orf198*, *LY86-AS1*, *PMP2* (also confirmed by RRA), we could see that effects for LY86-AS1 were related to analysis platform, PMI, and percentage of participants with schizophrenia in all brain subset (Data S17). The effects for PMP2 were related to PMI in the all brain subset and the in cortical subset and also to the percentage of male participants in the cortical subset. Other genes from this list were either not analyzed (n_cohorts_ < 10) or non-significant for any coefficient.

### Comparison of brain meta-analysis with blood transcriptome and PSY proteome

The blood data were not included in the RE meta-analysis due to differences in sample sources (blood and brain tissues). The meta-analyses were based on brain tissue data, but we compared the findings with the analysis of blood transcriptomics in the cohort GSE247998 (Data S18).

Without covariate adjustment, probe-level blood direction from genes detected from all brain regions showed 86 genes (28.01%) with all negative direction, 137 genes (44.63%) with all positive direction, and 84 genes (27.36%) not detected or analyzed. Among the analyzed genes, only 7 (2.3%) were nominally significant also in the blood. Directional matching revealed 129 genes (57.85%) with matched directions with brain tissue, and only *SOX5, SERPING1, and NTAN1* were both matching in direction and significant in both blood and meta-analysis. In cortical and prefrontal subanalyses, we observed comparable percentage overlaps with blood, though lower than in all brain analysis (Data S18). Only two genes *ZC3H12B* and *NEDD4L* matched between blood and brain while being nominally significant in cortical analysis and seven genes matched in a similar setting in DLPFC (Data S18). Effect sizes (log_2_FC) were not correlated between blood and brain (Fig. S5).

Interestingly, covariate adjustment slightly increased (~11.9% on average for every tissue subset) the number of directionally matched hits between blood and brain. However, it had almost no effect on the number of significant directionally matched genes, and only four genes in all brain analysis, two genes in the cortical analysis, and four genes in DLPFC reached this criterion. SVs had almost no effect on matching with blood (~4% increase on average for all tissue subsets). However, it substantially increased the number of significant matches, reaching 4 genes for all brain analysis, 8 genes for the cortical analysis and 20 genes for DLPFC (Data S18).

Compared with the proteome results from the blood-based PSY cohort (Data S4), three proteins (RBKS, CCL27, AKT1S1) showed nominal significance in analyses without adjustment for antidepressant medication. After adjustment for antidepressant medication, one protein (PLA2G10) retained nominal significance. None of the PSY-detected proteins were supported by results from any of the meta-analyses.

### Cell deconvolution analysis and cell-specific meta-analysis

**Fig. 5:**
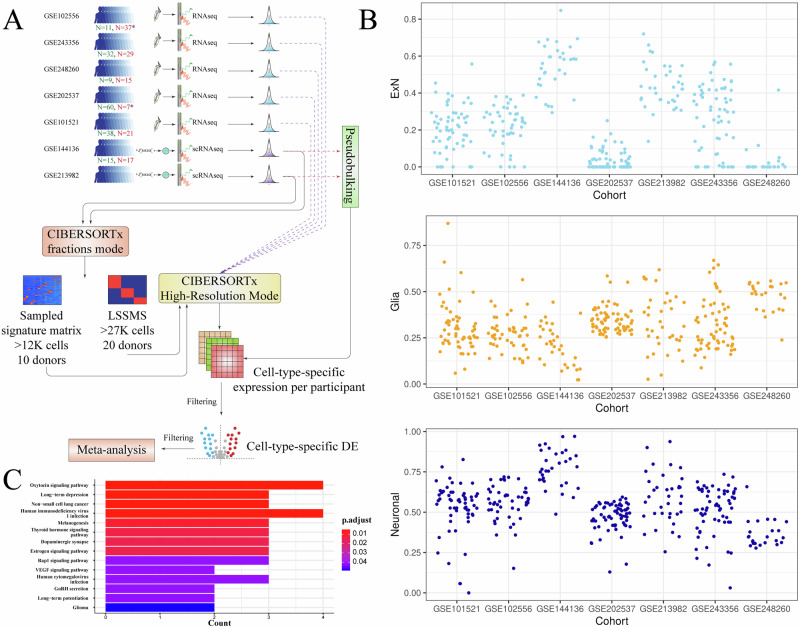
Cell-deconvolution meta-analysis. **A** This figure shows a flowchart of expression deconvolution meta-analysis in the present study. **B** This figure shows estimated proportions of three cell types (excitatory neurons ExN, broad neuronal subtype, and broad glial subtype) across RNAseq datasets. Cohorts GSE144136 and GSE213982 contain real proportions estimated directly by counting cell phenotypes. **C** This bar chart shows KEGG pathway enrichment for genes obtained in meta-analysis for excitatory neurons ExN. X-axis corresponds to gene count in the samples, whereas y-axis corresponds to the pathway category. Color of bars shows *p*-values after correction for multiple testing with FDR. DEs, differential expressions; LSSMS, large sampled signature matrix simplified.

Given the availability of single-cell reference data from GSE144136 and GSE213982, and computational tools, such as CIBERSORTx, it is interesting to investigate the relationship between cell-type specific expression and the suicide phenotype (Fig. 5A). The first step was to construct signature matrices from specified datasets (Data S1 1.12). LSSMS demonstrated good reconstructions of broad cell proportions (Fig. S6) with Spearman correlations above 85% for LSSMS. The sampled signature matrix demonstrated good reconstruction for major cell subtypes, however, failed to estimate proportions for oligodendrocytes and their precursor cells, as well as cells with mixed expression (Fig. S7). Subsequent evaluation of high resolution expression showed modest expression reconstruction for glial cells (Rp ~ 61%), and good reconstruction for neuronal cells (Rp ~ 93%) in LSSMS, whereas sampled signature showed good reconstruction (Rp ~ 79%) only in excitatory neurons (ExN) (Fig. S8-S9).

Subsequent cell-specific meta-analysis was only performed at simplified grouped levels (neuronal and glial) as well as on a cell-specific level only for excitatory neurons (Data S19). Cell proportion reconstruction identified variable cell proportions across RNAseq datasets (Fig. 5B). As cell expression imputation may be inaccurate on completely new cohorts, we only considered genes that passed nominal significance in at least one real single-cell cohort (GSE144136, GSE213982). ExN and neuronal analyses identified more nominally significant genes, reaching 47 and 90 hits, respectively. Glial cell sub-analysis identified 12 genes. Most genes failed to be analyzed as they did not meet imputation accuracy requirements from CIBERSORTx, did not pass primary significance in GSE144136, GSE213982 or did not reach required imputation accuracy in at least 5 datasets. No genes were significant at the FDR level. However, we found several genes, and in particular *GRB14*, *LY86-AS1*, *PLAAT1*, *LRRC9*, *CYB5R3*, and others that were in nominal agreement with primary bulk meta-analyses and are related to neuronal cell types. Subsequent enrichment analysis of these genes (Fig. 5C) indicated overrepresentation of depression-related KEGG pathways in ExN. No other enrichments met FDR significance thresholds in cell-specific analyses (Data S19).

### Gene set enrichment analysis and classification

We performed similar enrichments on the differentially expressed genes in each bulk meta-analysis (Data S20). In the analysis of all brain regions, one FDR-significant pathway related to cytoplasmic translation was identified without covariate adjustment, while nominally significant pathways associated with non-specific translation processes were observed with covariate adjustment. In cortical regions, glial cell-related pathways were identified after covariate adjustment, whereas pathways linked to oxidative stress and hypoxia were nominally significant without adjustment. In the DLPFC, over 100 FDR-significant pathways were identified without covariate adjustment, encompassing processes such as vascular development and transport, signal transduction, neural functions, amino acid metabolism and others (Fig. S10). Cellular location enrichment in DLPFC without covariates indicated glial cell projections, endosomes, as well as synapses (Fig. S11). With covariate adjustment, nominally significant pathways included those related to proliferative and immune processes, among others.

**Fig. 6:**
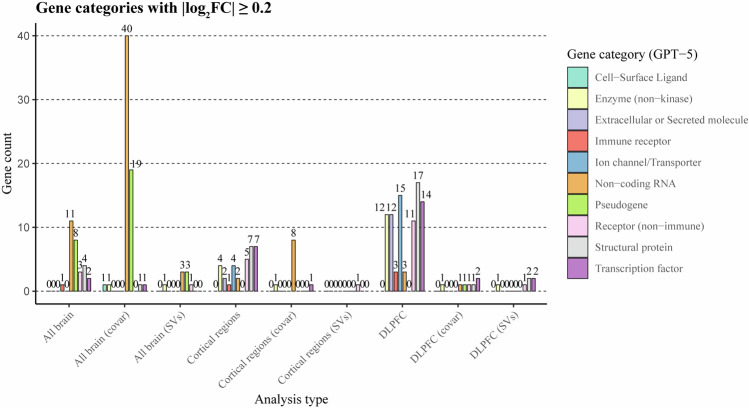
This figure shows GPT-5 large language model classification of genes with meta-estimated absolute log_2_FC ≥ 0.2 (P_nominal_<0.05). Prompt to the model is specified in the Data S1. Gene categories were suggested in the prompt and the model was tasked to assign one category for each gene symbol individually. Then classification was manually inspected for correctness and 5 entries were reclassified. Classification of the genes was performed on September 5 and December 2, 2025. X-axis indicates the type of meta-analysis, whereas the Y-axis shows gene counts per category.

To further understand biological properties of identified genes, we classified the set of meta-significant genes using GPT-5 LLM with manual curation and observed noticeable representation of secreted molecules, enzymes (non-kinase), immune receptors, kinases, ligands, non-coding RNA, pseudogenes, non-immune receptors, structural proteins, and transcription factors (Fig. 6). In the analysis of all brain regions, with and without covariate adjustment, non-coding RNA emerged as the most commonly enriched gene category. In cortical regions, structural proteins dominated without covariates, while non-coding RNAs were most common with covariates. In the DLPFC, different proteins were prominent without covariates. Surrogate variable analysis reduced overall effect sizes so only a few genes were classified with absolute log_2_FC ≥ 0.2.

## Discussion

This study presents a large meta-analysis of gene expression studies on suicide, utilizing publicly available postmortem brain data from a wide array of transcriptomic datasets across various brain regions and a blood-based sample. By comparing suicide cases with controls, primarily in the dorsolateral prefrontal cortex (DLPFC) and other cortical regions, our findings offer insights into the molecular landscapes associated with suicide. As shown in Fig. 3, several genes were significant in all three meta-analyses without covariates, including *PMP2, DEPP1, SOX9, CX3CR1, GPR34*, and *P2RY12*, highlighted in the orange dashed area. The blue dashed area indicates genes that were significant in meta-analyses with and without covariates across all brain tissues, such as *RP5-837J1.4, AC159540.14, DNM1P47, AC004158.2, EEF1A1P30*, and *RP11-339B21.8*. Gene classification analysis revealed diverse biological processes linked to the differentially expressed genes across brain regions, suggesting potential roles for non-coding RNA, structural proteins, and immune receptor processes in the pathology of suicide.

In this work, we prioritized performing analysis using a multiverse strategy where we implemented several possible analysis directions. Many decisions can potentially affect the outcome of analysis, such as inclusion of phenotype covariates or surrogate variables in the models. We also observed relatively different results if we change the main methods of meta-analysis from the RE model to RRA (though we did observe overlapping markers, such as PMP2 and others). Varying degrees of tissue specificity restriction also changed obtained gene sets, however, estimated effects remained somewhat consistent (Fig. 4D–F, Fig. S2). Some genes also overlapped between brain regions. However, it may be due to many studies focusing on DLPFC samples. Nonetheless, we could see direct support in the literature for genes obtained in unadjusted models and SVs.

One of the top upregulated genes overlapping across all regions in the unadjusted analyses and SV-based analyses is *P2RY12* (Figs. 2 and 3). Purinergic receptor 12 (*P2RY12*) is noteworthy due to the growing body of literature highlighting its role in microglial function [66], as well as the proposed link between suicide, glial cells, and purinergic signaling pathways, including *P2RY12* [67–73]. *P2RY12* belongs to the P2Y family of metabotropic G protein-coupled purinergic receptors and is highly expressed in microglia compared to other brain and myeloid cell types. Notably, *P2RY12* expression is reduced upon immunogenic microglial activation [74], in vitro tissue culturing [75] and is decreased with aging [76]. *P2RY12* has been shown to influence microglial migration, activation, and neuronal activity [77, 78]. Several studies have reported elevated P2RY12 mRNA in individuals who died by suicide. For instance, one study observed a more than 50% increase in *P2RY12* mRNA specifically in suicidal individuals with schizophrenia (SCZ) in the anterior cingulate cortex, suggesting that disruptions in purinergic signaling may be linked to suicidality, potentially independent of the primary mental illness [68]. It was further proposed that elevated *P2RY12* expression in individuals who died by suicide could reflect a reduction in ADP inactivation, as P2RY12 activity is inhibited by ADP during purine metabolism [68]. Another study found that *P2RY12* levels were approximately halved in non-suicidal patients with major depressive disorder (MDD), but not in suicide completers, compared to controls, suggesting that these effects could relate to platelet coagulation, given that *P2RY12* is a critical regulator in platelet aggregation. In cases of suicide, increased platelet aggregation may result from capillary injury activating the complement system [67]. In a third study, *P2RY12* expression was elevated in suicide completers with bipolar disorder (BD) compared to non-suicidal patients, which may indicate microglial activation associated with suicidal behavior [69]. All of these findings align with our meta-analysis results, which consistently showed upregulated levels of *P2RY12* in suicide cases compared to controls.

All the genes significant both with and without covariate adjustment across all brain tissues, as shown in the blue dashed area in Fig. 3, encode long non-coding RNAs (lncRNAs). LncRNAs are RNA molecules longer than 200 nucleotides that have little or no protein-coding potential [79]. They are transcribed by RNA Polymerase II and undergo the same maturation processes as protein-coding mRNAs [80]. Emerging research has revealed that lncRNAs are highly versatile in function. They can modify chromatin structure [80, 81], act as “sponges” to inhibit microRNA functions [82], provide docking sites for proteins [83], regulate mRNA transcription as activators or suppressors, and influence RNA splicing patterns [84]. In the brain, lncRNAs are particularly involved in developmental processes [85], such as the spatiotemporal control of pluripotent stem cell proliferation and differentiation [86, 87]. Research into the role of non-coding RNAs in psychiatric disorders is expanding. Studies have linked altered lncRNA expression to suicidal behavior [88–91], though the limited sample sizes and number of studies necessitate further validation. Acting as intermediaries between genetic variation and phenotype, lncRNAs influence biological pathways through epigenetic regulation of gene expression. Although the study of lncRNAs in psychiatric disorders is still in its early stages, understanding their influence on disease onset and progression could not only lead to more refined and objective diagnostic tools but also identify novel therapeutic targets. These preliminary findings are promising and highlight the need for continued investigation.

We also found that the expression of four additional genes, *SOX9, CX3CR1, GPR34*, and *PMP2*, differed significantly in suicide cases compared to controls, displaying consistent changes across all unadjusted meta-analyses (Figs. 2 and 3, highlighted in the orange dashed area). The SOX9 gene was downregulated in suicide cases, aligning with previous findings [92–94]. *SOX9* is an astrocytic marker in the brain and a B cell marker in blood, previously shown to be decreased in the frontal cortex in MDD [26] and in depressed suicides [92]. A study by Ernst et al. [93] found that *SOX9* was significantly downregulated in post-mortem DLPFC samples of suicide completers. Given its role as a transcription factor in astrocytes, this finding suggests that astrocytes may have a potential role in the biological processes associated with suicide. Interestingly, previous studies have demonstrated that specific astrocyte-derived neurotrophic factors, such as VEGF and BDNF, are linked to suicidal behaviors, with VEGF playing a role in regulating neurogenesis and astrocyte differentiation [95–97]. However, the present analysis did not identify contributions of these genes to suicide with effect sizes nearing zero regardless of covariates and tissues. However, previous data on antidepressants like fluoxetine indicate that increasing the expression of astrocyte-derived neurotrophic factors may help restore the trophic and metabolic support to neurons in major depression [98], warranting further investigation.

Conversely, the *CX3CR1* gene expression was upregulated in suicide cases compared to controls in the unadjusted meta-analyses. One study reported a significant increase in *CX3CR1* expression in suicide patients with schizophrenia (SCZ) compared to those who died of other causes. Among suicide victims with SCZ, *CX3CR1* expression was higher compared to SCZ patients who did not die by suicide. As a chemokine receptor, the ligand of *CX3CR1*, chemokine (C-X3-C motif) ligand 1 (*CX3CL1* or fractalkine), has not been directly associated with suicide or SCZ before, though it has been linked to mood disorders [99]. Another study found dramatically increased *CX3CR1* expression in patients with moderate to severe MDD [100], who were at high suicide risk. Together with the upregulation of *P2RY12*, both *CX3CR1* and *P2RY12*, which are homeostatic microglial genes, may indicate dysregulated and activated homeostasis in suicidal individuals [101].

Additionally, two other genes, *GPR34* and *PMP2*, showed altered expression in suicide cases in our meta-analysis. *GPR34*, a member of the G protein-coupled receptor family, was upregulated in suicide cases compared to controls. *GPR34* plays a fundamental role in cellular survival, differentiation, and apoptosis [102, 103] and exhibits pro-inflammatory and pro-tumor effects [104, 105]. It is highly expressed in immune cells and responds to toll-like receptor stimulation [106]. Immune responses were found to be altered in mice with *GPR34* deficiency [107]. Previous research has identified *GPR34* as a signature gene in both mouse and human microglia [108–110], and microglia lacking *GPR34* showed altered cell morphology [111]. In Alzheimer’s disease, high hippocampal *GPR34* levels have been linked to microglial activation and inflammation, potentially leading to neuroinflammation and cognitive impairment [112]. No prior studies have examined *GPR34* in relation to suicide, but our results provide evidence of its upregulation in suicide cases. Given its association with microglial activation and inflammation, the elevated *GPR34* levels in our meta-analysis suggest that neuroinflammation, possibly mediated by *GPR34*, may play a role in the suicide process, warranting further investigation. Interestingly, a study on a schizophrenia-like model in rats found that treatment with ketamine, a drug with well-documented anti-suicidal effects [113], affected three of the genes we identified: *P2RY12, CX3CR1*, and *GPR34* [114]. These genes, along with others associated with astroglial and microglial cells, are linked to immune-like processes that mediate synaptogenesis and neuronal plasticity. In theory, it could both highlight the importance of these genes in ketamine treatment effect or could also indicate potential undisclosed ketamine use in the suicidal individuals from the analyzed cohorts.

Finally, the *PMP2* gene, which codes for Peripheral Myelin Protein 2, a small 14 kDa protein in the fatty acid binding protein family (FABP), was downregulated in suicide cases. PMP2 is an abundant peripheral myelin protein involved in lipid trafficking, remyelination, myelin sheath stability, membrane stacking, and lipid transfer [115–118]. Mutations in *PMP2* have been linked to demyelinating Charcot-Marie-Tooth neuropathy [116, 119–121]. A recent meta-analysis reported decreased *PMP2* expression in the prefrontal cortex (PFC) and DLPFC of suicide cases compared to controls, consistent with our findings and supporting a link between reduced *PMP2* levels and suicide [19].

Unfortunately, our single-cell deconvolution analysis was not able to analyze cell-specific expression of the majority of genes due to lack of available RNAseq cohorts and lack of imputation accuracy for some genes. However, we could identify that neuronal subset points to most genes that are supported by several bulk tissue analyses. Interestingly, the enrichment of the ExN meta-analysis highlights depression-related signaling, primarily through *GNAO1*, *PRKCG*, and *MAP2K2*. Interestingly, decreased expression of *PRKCG* was directly related to depression and suicide [122], and matches the direction in our study.

While the overall sample size of our meta-analysis is relatively large for a gene expression study in brain samples, our findings should be interpreted with several limitations in mind. The suicide data were derived from studies with varying designs and underlying conditions leading to suicide. Since we relied on publicly available datasets, detailed participant characteristics were often unavailable. Consequently, we were limited regarding adjusting models for potential confounding factors such as psychiatric diagnoses, medication use, smoking, childhood trauma, recent life stress, psychiatric and medical comorbidities, substance use, environmental exposures, or time of death, among many others [123]. We applied a complete-case approach where covariates were available. However, this strategy reduces power and may increase bias. Different levels of postmortem intervals, brain pH, or RNA integrity across different studies may distort transcript abundance estimates and limit comparability across studies. Different technological platforms may introduce batch effects and limit comparability of even relative measurements such as log_2_FC. Additionally, some study designs in the primary datasets did not allow distinction between suicide and associated MDD. Therefore, it is difficult to establish whether identified genes are specifically related to suicide or related psychiatric diagnoses. The majority of the postmortem brain gene expression data came mainly from brain collections in North America, potentially limiting the generalizability of our findings to other populations. As with other association studies, our gene expression findings should not be interpreted as evidence of causation. Additionally, the lack of power when multiple testing is considered and the use of nominal p < 0.05 could increase the rate of false positives. Lack of power also limits interpretability of moderator analyses, as these typically require large numbers of primary studies. Even though prior cohorts were selected without a hypothesis regarding specific genes, potentially reducing positive publication bias common for meta-analyses, it is still possible that studies had this or other biases influencing the estimates. Even though we tested signature matrices on validation samples, it is possible the cell deconvolution was not accurately reconstructing gene expression of bulk RNA datasets, and together with nominal significance, this renders cell-specific analysis very exploratory.

However, the use of multiple cohorts to identify reproducible associations enhances the likelihood of uncovering biologically relevant findings rather than spurious results that may not generalize between studies. Furthermore, we performed sensitivity analyses to see how the identified genes are dependent on the covariate adjustment, model selections, as well as tissue specificity. Additionally, cell-deconvolution meta-analysis offers first insights into how specific genes are related to suicide in specific cell types and how they are consistent across cohorts. Combining our gene expression findings with complementary research approaches, such as genome-wide association studies, epigenomic studies, and preclinical models, can strengthen the evidence for the involvement of specific genes and pathways in the biological mechanisms underlying suicide.

## Conclusion

In summary, our meta-analysis serves as a hypothesis-generating bioinformatic exploration of publicly available expression datasets using several analysis strategies. Many of our findings are very consistent with existing literature on suicide transcriptomics, identifying *PMP2*, *SOX9*, *CX3CR1*, *GPR34*, *P2RY12*, and lncRNAs, as well as other genes, as plausible molecular targets for future validation studies. This study advances our understanding of the complex molecular architecture of suicide.

## Supplementary information

Data_S1

Fig_S1_S11

Data_S2_S20

## Data Availability

This analysis is based on open-access transcriptomic datasets available at GEO.

## Code availability

The source code for all stages of the work is publicly-available at https://github.com/AleksandrVSokolov/suicide_meta.
