# Supplementary material for: Identification of suicide brain transcriptomic signatures using meta-analysis of multiple cohorts
Source: Transl Psychiatry. 2026 Mar 31;16:222. doi: 10.1038/s41398-026-03978-8 (PMC13039836; doi:10.1038/s41398-026-03978-8)
Supplement: Supplementary file 1 — Data_S1 [file 41398_2026_3978_MOESM1_ESM.pdf]

Supplementary Materials (Data S1) for

Identification of suicide brain transcriptomic signatures using meta-analysis of multiple cohorts

Sokolov A.V., Lafta M.S., Jussi Jokinen and Schioth H.B.*

***Corresponding author. Email: Helgi.Schioth@neuro.uu.se or Helgi.Schioth@uu.se**

This PDF file includes:

Supplementary text

Descriptions Figures S1 to S11

Descriptions for Data S2 to S20

References

Other Supplementary Materials for this manuscript (not embedded in this file) include the following:

- Fig S1 - Fig S11

- Data S2 - Data S20 (compressed as .7z archive).

1. Supplementary methods **2**
1.1 Ethics declaration 2
1.2 Cohort identification 3
1.3 Cohort descriptions 3
1.3.1 The PSY cohort 3
1.3.2 Cohort GSE208338 4
1.3.3 Cohorts GSE5388 and GSE5389 4
1.3.4 Cohort GSE66937 5
1.3.5 Cohort GSE199536 5
1.3.6 Cohorts GSE92538 U133A and GSE92538 PLUS2 5
1.3.7 Cohort GSE102556 6
1.3.8 Cohort GSE243356 6
1.3.9 Cohort GSE248260 6
1.3.10 Cohort GSE247998 7
1.3.11 Cohort GSE202537 7
1.3.12 Cohort GSE101521 7
1.3.13 Cohorts GSE144136 and GSE213982 8
1.4 Differential proteomic profiling in the PSY 8
1.5 Transcriptome array data preprocessing 9
1.6 Bulk RNAseq data preprocessing 9
1.7 Single cell RNAseq data preprocessing 10
1.8 Differential expression analysis in the individual cohorts 10
1.9 Probe and transcript mapping to gene symbols 13
1.10 Meta-analysis of differential expression changes 13
1.11 Sensitivity analyses and moderator analysis 14
1.12 Cell deconvolution analysis 16
1.13 Gene enrichment analysis and classification 18
2. Descriptions for supplementary figures **19**
3. Descriptions for supplementary materials **22**
4. References **26**

1. Supplementary methods

This section provides a detailed description of the methods. All of the associated analysis code is publicly available at https://github.com/AleksandrVSokolov/suicide_meta.

1.1 Ethics declaration

The current work uses data from 15 public-access and one domestic cohort. The Psychiatric Health in Adolescent Study (PSY cohort) was performed in Uppsala, Sweden. The study was approved by the Regional Ethics Committee of Uppsala. All participants gave their written informed consent for participation. Data from 15 public-access cohorts has been

obtained from Gene Expression Omnibus (GEO) [1]. The use of data from GEO was approved by the Regional Ethics Committee of Uppsala. These studies were also approved by their corresponding national ethical review committees. Further information is available in the corresponding GEO records and/or in the initial publications.

1.2 Cohort identification

The identification of suicide cohorts has been performed on July 17th, 2024, querying the GEO repository with the two following search terms:

1. ("suicide"[MeSH Terms] OR suicide[All Fields]) AND ("human"[Organism] AND ("Expression profiling by array"[Filter] OR "Expression profiling by genome tiling array"[Filter]))
2. ("suicide"[MeSH Terms] OR suicide[All Fields]) AND ("human"[Organism] AND "Expression profiling by high throughput sequencing"[Filter])

To be included in the analysis, GEO cohorts were required to pass all of the following criteria:

1. A study must have confirmed suicide cases. Studies on psychiatric diagnoses, such as major depressive disorder, were not analyzed without clear identification of individuals who committed/attempted to commit suicide.
2. A dataset must have at least 5 suicide cases
3. Expression signals or counts must be available at the resolution of the individual participants and individual transcripts.
4. Study design and study methods must allow comparison between suicide and non-suicide groups.
5. The study must have been conducted on human subjects and must involve human samples and not their derivatives (such as cell cultures).

The PSY cohort was included as a separate analysis (not a part of meta-analysis) since the research group had access to it.

1.3 Cohort descriptions

Demographic data for the PSY cohort is available in Data S2. Demographic data for all GEO cohorts could be found in the Data S3.

1.3.1 The PSY cohort

The Psychiatric Health in Adolescent Study (PSY cohort) was performed in Uppsala, Sweden. This cohort includes adolescents from schools in Uppsala, Sweden recruited at ages 14-17 on the first visit. Participants passed through a series of psychiatric questionnaires to evaluate their mental health. Suicide-related health evaluation was performed during the second visit (approximately 1 year after recruitment), utilizing SUAS-S questionarrie [2]. Participants were classified to the risk group if they had a score greater or equal to 29. The study sample included 378 participants classified as normal and 26 participants classified as suicide risk group. The whole blood sample was collected during the second visit to obtain serum and plasma. Plasma samples were further used for proteomic quantification with Olink proteomic assays.

Proteomic profiling was performed with the Olink Neuro Exploratory Panel involving 92 proteins (Neuro Exploratory Panel of Olink Proteomics AB, Uppsala Sweden) in two batches. Proximity Extension Assay was performed according to the manufacturer's instructions with default regents. A sample of 1 µL of EDTA-containing plasma and a 3-µL incubation mix was incubated overnight at 8°C. Then a 96-µL extension mix with PEA enzyme and PCR reagents was added and incubated at room temperature for 5 min. Afterwards, an extension reaction in a thermal cycler was performed followed by 17 cycles of DNA amplification. Once the antibody probes bound to the targeted protein and the attached DNA oligonucleotides were in close proximity, the oligonucleotides hybridized and were extended by enzymatic polymerization. The oligonucleotide templates were then amplified and quantified using real-time polymerase chain reaction. Protein levels are quantified as Normalized Protein Expression (NPX) values on log2-scale [3]. All assay procedures were performed at Affinity Proteomics Uppsala SciLifeLab in Sweden.

1.3.2 Cohort GSE208338

The cohort GSE208338 was collected at the Victorian Institute of Forensic Medicine, Australia. The study data encompasses 169 post-mortem dorsolateral prefrontal cortex samples from adult subjects aged 18-87 years with either schizophrenia (SCZ) (n = 68), major depressive disorder (n = 24), BD (n = 15), and controls (n = 62). Based on information from GEO, total RNA was isolated from gray matter samples [4]. Among included participants, 59 were in the suicide group, whereas others were the non-suicide group. The data for diagnostics, clinical characteristics, and pharmacological profile was obtained utilizing the Diagnostic Instrument for Brain Studies (DIBS). Further details on sample preparation and characterization of participants are available in the original publications [4, 5]. Phenotypic data included information on age, sex, tissue pH, post-mortem interval, RNA integrity number (RIN), psychiatric diagnosis (SCZ/MDD/control), suicide death, type of death (violent/natural/non-violent), and the data on four genetic principal components and one surrogate variable that were used to adjust for heterogeneity in the statistical analysis. Gene expression profiling of the cohort GSE208338 was performed with Affymetrix Human Exon 1.0 ST v2 Arrays and the library was prepared with "Affymetrix synthesis and labelling kit" [6]. We used already available preprocessed data in the analysis as it was adjusted for five known batches that were not reported in the phenotype data on GEO.

1.3.3 Cohorts GSE5388 and GSE5389

The cohorts GSE5388 and GSE5389 were obtained from Stanley Medical Research Institute, US and included post-mortem brain samples from dorsolateral prefrontal cortex (GSE5388) and orbitofrontal cortex (GSE5389). The initial publication and GEO repositories do not specify regarding gray/white matter content. The samples were obtained from 61 participants (31 controls and 30 bipolar) in GSE5388 and from 21 participants (11 controls and 10 bipolar) in GSE5389. Samples included in the prefrontal cortex dataset and orbitofrontal cortex dataset were derived from different individuals, and thus are independent biological replicates. One control sample GSM123233 has been discarded from the analysis as it contained a distinct RNA degradation plot slope compared to other participants. Participants were also characterized regarding their suicide status, thus 12 participants were referred to the suicide group in GSE5388 and six participants were in the suicide group in GSE5389. Additional phenotype information included sex, age, brain tissue pH, post-mortem interval, fluphenazine treatment dose, valproate treatment (binary), lithium treatment (binary), electroconvulsive therapy (binary), alcohol abuse rating scale, and drug abuse rating scale. Data also contained information on duration of illness, age of onset, and tissue

collection side of the brain, however these covariates were not used as deemed not relevant by authors and were also not significantly associated with disease phenotypes in the original publication [7]. Gene expression profiling in GSE5388 and GSE5389 was performed with Affymetrix Human Genome U133A Array and libraries were prepared with "BioArray HighYield RNA transcript Labeling Kit". Please refer to the original publication for further information regarding sample preparation [7].

1.3.4 Cohort GSE66937

The cohort GSE66937 was collected from the Douglas Bell Canada Brain Bank and included samples 15 participants (9 suicide group) from four tissues: amygdala, hippocampus, thalamus and prefrontal cortex. No information was available regarding gray/white matter content in the samples. The data provided on GEO included only RIN besides suicide status information. The initial publication contained demographic data on sex, PMI, age, cause of death, psychiatric diagnosis, and toxicological information. However, these covariates were not available on GEO or in the supplementary materials (with no matching identifiers to GEO)[8], and thus were not used in the analysis. Additionally, the initial article reports demographics on 20 participants. Transcriptome libraries were prepared with "WT PLUS reagent kit" and profiles were analyzed with Affymetrix Human Transcriptome Array 2.0. We observed different distributions in the expression signals obtained in the study depending on the hybridization year (2013 and 2014). Even though the distribution was corrected after data normalization and preprocessing, we decided to add a batch covariate reflecting hybridization year to phenotype data and used it in the analysis. Further information on sample acquisition, preparation, and experimental procedures is available in the initial article for this cohort [8].

1.3.5 Cohort GSE199536

The study GSE199536 contains 20 post-mortem brain samples obtained from the Douglas Bell Canada Brain Bank, Canada. In this study, habenula samples were prepared from 10 MDD suicide subject and 10 controls. Phenotype information was collected from related individuals best acquainted with the deceased via a series of structured interviews [9]. Phenotype data on GEO included information on sex and and suicide group. All subjects are male Caucasians. Additional phenotype information was collected from the study supplementary materials (Table S1) and included age, post-mortem interval, and tissue pH (measured in cerebellum). Expression levels were analyzed with Affymetrix Human Gene 2.0 ST Array on libraries prepared with "GeneChip WT Terminal Labeling and Controls Kit". Please refer to the original publication for information on wet lab procedures [9].

1.3.6 Cohorts GSE92538 U133A and GSE92538 PLUS2

The cohort GSE92538 was obtained by the Pritzker Neuropsychiatric Disorders Research Consortium at several research sites located in the US [10–13]. The initial sample contains dorsolateral prefrontal cortex (DLPFC) tissue from 172 subjects. The tissue collection was primarily performed in such a way to predominantly include gray matter [12]. Phenotype data contains information on participant sex, age, post-mortem interval, quality control batch, ethnicity (race), suicide death, agonal factor score (AFS), and tissue pH (measured in cerebellum). We dichotomized the agonal factor score into two groups: AFS=0 and AFS >=1 that indicate absence and presence of the agonal factors, respectively [14]. Gene expression analysis was performed by two arrays: Affymetrix Human Genome U133A Array and Affymetrix GeneChip Human Genome HG-U133 Plus 2 Array. The exact library

kit was not specified and listed as "standard Affymetrix protocol" [12]. The data at GEO contains 363 records, which include duplicated measurements of the same individuals at several research sites, as well as measurements of the same individuals utilizing both arrays. For the purpose of the current work, we considered each individual only once. All duplicated measurements were discarded after data preprocessing. The samples analyzed by both platforms were discarded from the Affymetrix Human Genome U133A Array dataset but kept in the Affymetrix GeneChip Human Genome HG-U133 Plus 2 Array dataset to avoid overlap. Thus, the resulting sample included 97 participants (17 suicide) in U133A data, whereas 75 samples (20 suicide) were kept in PLUS2 data. Please refer to initial publications for information on experimental procedures [10–13].

1.3.7 Cohort GSE102556

The cohort GSE102556 was obtained from the Douglas Bell Canada Brain Bank, Canada and contains post-mortem samples from six tissues: orbitofrontal cortex (OFC; BA11), dorsolateral PFC (BA8/9; dlPFC), cingulate gyrus 25 (BA25; cg25; vmPFC), anterior insula (aINS); nucleus accumbens (NAc) and ventral subiculum (vSUB). There are 48 samples for OFC, 48 samples for dlPFC, 28 samples for vmPFC, 48 samples for aINS,48 samples for Nac, and 43 samples for vSUB. Tissue was obtained from the same individuals (48 in total). All participants were Caucasians of French–Canadian descent [15]. Phenotype data on GEO contains information on sex, age, cause of death (accident, natural, or suicide), diagnosis (control or MDD), post-mortem interval, tissue pH, alcohol intake, drug use, drug type, medication use, medication type, and smoking. For the purpose of this project, we generate a suicide grouping variable, that was derived from cause of death and defined either as suicide or non-sucide. Gene expression profiling was performed via RNA sequencing with library prepared utilizing "ScriptSeq Complete Gold Kit" and sequencing performed with Illumina HiSeq2500 platform with 50 bp paired-end layout. We obtained sequencing reads from NCBI Sequence Read Archive (SRA). Further information could be found in the original study article [15]. One participant (SRA SRR5961809) was excluded from the data due to low mapping percentage compared to other samples.

1.3.8 Cohort GSE243356

The cohort GSE243356 was created with samples from the New York Psychiatric Institute and Columbia University Psychiatric Disorder Macedonian/NYSPI Brain Collection [16]. The study includes 61 temporal cortex samples (29 suicide) obtained from BA20 and BA36 regions. Samples represented gray and white matter mixtures. Phenotype data on GEO contains only information regarding a group (suicide or control). The groups did not differ significantly for sex and age according to the original study [16]. Gene expression profiling was performed with RNA sequencing, utilizing "KAPA RNA HyperPrep Kit" for library preparation and Illumina NovaSeq6000 sequencing platform with 100 bp, paired-end configuration. Sequencing reads were deposited on SRA and obtained for the current analysis. Details on wet lab procedures are available in the initial article [16].

1.3.9 Cohort GSE248260

The study GSE248260 was conducted at the New York State Psychiatric Institute. The cohort consists of 15 suicide decedents and 9 non-psychiatric controls. The sample for every participant represents post-mortem tissue from the ventral white matter (BA 47) [17]. Phenotype data from GEO contains characteristics on age, sex, suicide status, and diagnosis (MDD or control). Diagnosis of MDD was fully related to suicide status. Supplementary

materials of the initial article provide additional information on RNA integrity, post-mortem interval, tissue pH, and toxicological reports on antidepressant, antipsychotics, benzodiazepines, and opioids. Further information could be found in the initial publication [17]. For the purpose of the current work, we created an additional binary factor indicating drug intake, which was defined as present if at least one toxicology report was positive. Expression profiling in this study was conducted utilizing Illumina HiSeq 2500 platform (paired-end, 50 bp layout) and raw reads were available on SRA. The initial RNA library was constructed with the "Truseq library preparation kit".

1.3.10 Cohort GSE247998

The cohort GSE247998 was also collected at the New York State Psychiatric Institute. The dataset includes 100 samples of peripheral blood from participants with reported history of suicide attempt (n=27), healthy controls (n=27), and MDD with no history of suicide attempt (n=50) [17]. All participants were alive and were evaluated via the Structured Clinical Interview for DSM-IV. Available phenotype data includes suicide attempt status, sex, age, diagnosis (MDD or no MDD), as well as suicidal ideation group (no,low, and high). Expression profiling in this cohort was performed with RNA sequencing, using Illumina HiSeq 2500 platform (paired-end, 50 bp layout) and raw reads were available on SRA.

1.3.11 Cohort GSE202537

The cohort GSE202537 was obtained through University of Pittsburgh Brain Tissue Donation Program and National Institutes of Health NeuroBioBank [18]. The study sample includes post-mortem samples from Nac (n=67, suicide n=7), caudate (n=70, suicide n=8), and putamen (n=69, suicide n=8). Tissues were collected from the same individuals. Phenotype characterization provides information on manner of death (accidental, natural, suicide, undetermined), sex, age, disease state (control, SCZ, BD), BMI, corrected time of death, sequencing library size, tissue pH, post-mortem interval, race (black/white), and RNA integrity. In this work, we created suicide binary factor that was present if the manner of death was equal to suicide and not present if otherwise. All samples with the unknown manner of death (2 per tissue) were excluded from analysis. The RNA library was prepared with the TruSeq Stranded Total RNA Sample Preparation Kit. Gene expression quantification was conducted with Illumina NextSeq 500 RNA sequencing platform and data was deposited on SRA. Please refer to the initial article for further details [18]. Three samples (SRA: SRR19147540, SRR19147610, SRR19147547) had the majority of unpaired reads (more than ⅔) and, thus, were excluded from the analysis.

1.3.12 Cohort GSE101521

The cohort GSE101521 was created at New York State Psychiatric Institute using the material from the brain collection of The Division of Molecular Imaging and Neuropathology [19]. Whole-exome sequencing dataset includes 21 subjects with MDD and suicide, 9 subjects with MDD and no suicide, and and 29 sudden death healthy controls. All post-mortem samples were obtained from the dorsolateral prefrontal cortex (BA9). Phenotype data includes sex, age, diagnosis, RNA integrity, post-mortem interval, and tissue pH. For the purpose of current work, we added a suicide binary factor that was present for 21 subject with suicide and absent for other participants. We also added a depression factor that was present for all participants with MDD and absent in controls. RNA sequencing for this study was performed with Illumina HiSeq 2500 (2x100) for whole-exome profiling and with Illumina MiSeq (1x50) for microRNA. RNA library was prepared with the "TruSeq Stranded Total

RNA Sample Preparation Kit". RNA sequencing data was deposited on SRA. Further details are available in the source publication [19]. In the current work, we only used data from HiSeq 2500 for comparability with other datasets.

1.3.13 Cohorts GSE144136 and GSE213982

Cohorts GSE144136 and GSE213982 were initially created separately but then reanalyzed by the same group [20, 21]. We used datasets from the second publication. The cohort GSE144136 was collected from the Douglas–Bell Canada Brain Bank and represents post-mortem gray matter samples from the dorsolateral prefrontal cortex of male participants. Initial dataset included 17 suicide cases and 17 controls. Runs for the male participant 25 did not pass quality control in the initial study and were discarded. Runs for the male participant 24 were split in two and were also excluded from the analysis. Two samples F19 and F5 were processed using a separate platform BGI DNB-seq technology other than all other female samples and were excluded from analysis. Cohort GSE213982 was obtained from the Douglas–Bell Canada Brain Bank and University of Miami Miller School of Medicine Brain Endowment Bank. This cohort represents female post-mortem dorsolateral prefrontal cortex samples. The GSE213982 dataset includes 18 cases and 18 controls. Deposited phenotype information for both datasets includes only suicide status. All participants in the male cohort of Caucasian descent. One female case subject and three female control subjects were Hispanic, two female control subjects were African American according to the initial publication. This data was not available on GEO and therefore not used for the analysis [21]. Gene expression profiling was performed in the single-cell RNA sequencing fashion, utilizing isolated nuclei from cells. Details on experimental procedures are available in the source publications [20, 21]. RNA libraries were prepared with Chromium Single Cell 3' Reagent Kits v2 and Chromium Next GEM Single Cell 3′ Reagent Kits v3.1 and sequencing runs were performed with Illumina HiSeq 4000 and Illumina NovaSeq 6000. For the purpose of current analysis and comparability of two scRNA-seq datasets, we used raw counts deposited at GSE213982 for both male and female cohorts. Male and female cohorts were analyzed individually. We estimated cell proportions for every participant for every cell type as cell count for individual cell type divided by a total sum of all cell types for a participant,

$$Proportion_i = \frac{Count_i}{\sum_{i=1}^{N} Count_i}$$

and these proportions were used as covariates in further analyses. N denotes a number of cell types.

1.4 Differential proteomic profiling in the PSY

Differential proteomic profiling in the PSY cohort was performed within the R programming language environment. We used R package *limma* that leverages the Bayes method to moderate expression variance during fitting linear models [22]. This package was initially developed for transcriptome arrays but is commonly used for Olink NPX data [23–26] which is also on a log2 scale similar to array data after RMA normalization [27]. Before the analysis, NPX data was corrected for batch effect using *ComBat* [28] from the *sva* R package. In the initial differential proteomic analysis, the SUAS risk group was used as the main predictor, and models were adjusted for sex and age. In the adjusted analysis, we added an additional predictor indicating antidepressant intake. Analyses were performed as

"complete case", and participants without clear identification regarding antidepressant intake were excluded. Standard *limma* pipeline with default parameters was applied, and *topTable* was used to obtain differentially expressed proteins. We included both raw p-values and false-discovery-rate-adjusted p-values.

1.5 Transcriptome array data preprocessing

Gene expression profiling was performed via transcriptome arrays in the following cohorts: GSE208338, GSE5388, GSE5389, GSE66937, GSE199536, GSE92538 (U133A), GSE92538 (U133 PLUS2). We used already available preprocessed data from GEO for GSE208338 as it was adjusted for batch effects that were not included in the phenotype data in GEO. In all other array-based cohorts, we prepared the data starting from raw .CEL files. Data preprocessing, filtering, and quality control procedures were performed in R (version 4.2.3). First, raw .CEL files were downloaded from GEO using the R package *GEOquery* [29]. Then, depending on the array, we imported .CEL files into the R environment, utilizing either the *affy* R package [30] (Human Genome U133A, Human Genome HG-U133 Plus 2) or the *oligo* R package [31] (Human Transcriptome Array 2.0, Human Gene 2.0 ST). Data import was performed with *ReadAffy* function in the *affy* R package, whereas *read.celfiles* was used in the *oligo* R package. After data import, individual array intensities were inspected with density plots and boxplots for consistency. In the cohort GSE66937, we observed two expression patterns that were related to hybridization year of the arrays. This batch effect was added as an additional covariate in the statistical analysis stage. In the cohort GSE92538 (U133A), we observed a relatively inconsistent distribution of intensities for some of the arrays. These arrays had median intensities either below 6.6 or above 10. As samples in GSE92538 (U133A) were analyzed by several arrays (technical replicates), we were able to discard all outlying arrays without losing participants. In other cohorts, expression patterns were deemed somewhat consistent and did not warrant sample filtering. After filtering based on intensities, the data was normalized using the RMA method (default parameters) [27] in all array-based cohorts. After normalization, array intensities were explored with density plots and boxplots for consistency again. All cohorts (including GSE66937) showed consistent expression patterns within studies after processing with RMA. We further explored arrays to see if there are any samples showing different RNA degradation patterns compared to other samples. One array in GSE5388 (GSM123233) demonstrated a distinct degradation slope compared to other samples, and thus was excluded from the analysis. Once data normalization is complete, we perform filtering of the samples based on analysis relevance, namely all duplicated measurements, technical samples, etc. are removed. This step is different for every cohort and not always required (see cohort description above). After last sample filtering, RMA-normalized intensities were used for differential expression analysis.

1.6 Bulk RNAseq data preprocessing

We processed data from RNAseq reads deposited on the NCBI SRA portal. First SRA toolkit https://github.com/ncbi/sra-tools was used to prefetch and obtain reads from SRA in the form of .fastq files. One read (SRR26854217) was not downloaded in the cohort GSE247998 through the SRA toolkit, and we used the European Nucleotide Archive instead https://www.ebi.ac.uk/ena/browser/home to obtain .fastq files. We performed quality control of the first few runs in every cohort to see if sequencing data was preprocessed, contains sequencing adapters or other issues. We used *FastQC* [32] and *fastp* [33] tools for quality control. After inspection of the first several runs in every cohort, we did not observe substantial read problems and presence of adapters. We also tested pilot runs to quantify gene expression counts with and without trimming and observed no substantial differences in

counts, which is consistent with previous observation [34]. Thus, read trimming was not added to the data preprocessing pipeline. Read alignment and expression quantification was performed with the *STAR* aligner [35] with enabled quantification mode with default alignment parameters for paired-end reads. Genome index for *STAR* was generated using the latest available assembly for hg19 (GRCh37.p13) at Ensembl. This assembly was chosen to enable better comparability with array-based data which was designed for hg19. We used primary assembly file .fa.gz: https://ftp.ensembl.org/pub/grch37/current/fasta/homo_sapiens/dna/Homo_sapiens.GRCh37.dna_sm.primary_assembly.fa.gz. For the genome annotation, we used GRCh37.87.gtf.gz annotation file: https://ftp.ensembl.org/pub/grch37/current/gtf/homo_sapiens/Homo_sapiens.GRCh37.87.gtf.gz.

These data preprocessing steps were performed sequentially using custom python script. Runs through the SRA toolkit and *STAR* were called via the python *subprocess* module. Analysis was performed within the *conda* environment on a Linux machine (Ubuntu 22.04.2 LTS). Once counts were obtained, corresponding data and associated mapping and quantification statistics from *STAR* were imported into R. We inspected mapping percentages and count statistics to identify potentially problematic samples. One run in GSE102556 (SRR5961809) was excluded due to low mapping percent. Three runs in GSE202537 (SRR19147540, SRR19147610, SRR19147547) were excluded due to low read counts compared to the rest of the samples. Mapping of other runs was deemed as being of sufficient quality. Obtained counts were further used in differential expression analysis.

1.7 Single cell RNAseq data preprocessing

In the datasets GSE144136 and GSE213982, read data was generated from single cell runs. As raw counts were available for every cell and gene and both datasets were preprocessed in a consistent manner [21], we decided to use these counts directly. Sparse matrix was imported in R via the readMM function from the *Matrix* package. Then the obtained matrix was imported into *Seurat* R package[36]. We used cell annotation and meta-data available from the initial publication [21] and its GEO record. Obtained single cell data was pseudo-bulked in two ways: by individual and by both cell and individual. To take into account differences in cell compositions, we estimated cell proportions for every individual. Runs for the male participant 25 were excluded from the count matrix in the initial study due to failed quality control. Runs for the male participant 24 were split in two for unknown reasons, and we also decided to exclude them for better comparability between samples. Obtained pseudo-bulk counts were further used in differential expression analysis.

1.8 Differential expression analysis in the individual cohorts

Differential expression analysis in the individual cohorts was performed using R package *limma* that uses the Empirical Bayes method to moderate expression variance during fitting linear models [22]. RMA-normalized signal intensities were used for analysis with *limma* in array-based cohorts. If a cohort had more than one tissue, limma-based analysis was performed separately for every tissue. In RNAseq cohorts, raw transcript counts were first imported to the *DGEList* object from the edgeR package [37] and filtered using the *filterByExpr* function. This function uses transcript filtering strategy for the specified experiment design matrix, keeping only those transcripts that have at least minimal acceptable gene count per groups of participants. This filtering is extensively described in the original publication [38]. After filtering, we calculated normalization factors for the DGEList

object. To enable limma-based analysis, the obtained counts in the DGEList object passed through voom transformation [39] implemented in *limma.* Then voom-transformed counts were used in the standard *limma* analysis pipeline. If an RNAseq cohort had counts from more than one tissue, count filtering, voom preprocessing, and limma-based analysis were conducted separately for every tissue. Differential expression analysis pipeline was performed using *limma lmFit* and *eBayes* with default parameters (after voom for RNAseq), and *TopTable* was used to obtain differentially expressed transcripts. We also added standard errors (SE) for log2 fold changes that were estimated directly from the *limma fit* object using instructions specified by a package developer on the package support forum: https://support.bioconductor.org/p/70175/.

As there are multiple possibilities to conduct statistical modeling [40–42], we performed differential expression analysis with *limma* in three strategies. The first strategy only considered suicide status (binary) as a predictor without adjustment for covariates. The second strategy included suicide status (binary) and covariates that were deemed as relevant for analysis by authors (see further rationale for the approach in section 1.10). The relevance of covariates was determined if it has the possibility to affect gene expression and outcome, whether it has a sufficient number of levels (for categorical variables), as well as how many samples may be excluded due to missing values. We performed all analyses as "complete case", and participants with missing information regarding included covariates were excluded. If a covariate showed full dependence on the suicide status or other predictors, it was omitted. The list of relevant covariates was different between the cohorts as different phenotype information was available for every study in the first place. If a study had no extra covariates for analysis, the results without covariates were used instead. The third strategy considered adjusting initial limma modes with suicide status (binary) and estimated surrogate variables (SVs) using R package *sva*. We adjusted for a maximum of five surrogate variables per dataset, considering the number of samples in cohorts. The number of estimated SVs was calculated using the function *num.sv* with method "leek" [43]. If this number yielded 0, no SVs were used and analysis was conducted only with suicide status. In RNAseq datasets, *num.sv* identified large numbers of suggested surrogate variables, therefore we used 5 SVs in each of these datasets.

Below we provide adjusted model descriptions for every cohort as well as number of used surrogate variables in the SV-adjusted analysis:

1. **GSE208338**. Suicide status (binary) was used as the main predictor. Models were adjusted for age, sex, tissue pH, post-mortem interval, RNA integrity number, four genetic principal components (all numeric), one surrogate variable for hidden batch effects, diagnosis, and type of death. One SV was used in the SV-adjusted analysis.
2. **GSE5388**. Suicide status (binary) was used as the main predictor. Models were adjusted for disease status, age, sex, tissue pH, post-mortem interval, fluphenazine treatment dose, valproate treatment (binary), lithium treatment (binary), and alcohol abuse rating scale. Electroconvulsive therapy had only one factor level after removal of participants with missing data on other predictors, and thus was not used. Drug abuse rating scale was not used due to a very high fraction of participants with missing information. Five SVs were identified and five were used in the SV-adjusted analysis.
3. **GSE5389**. We used the same predictors as in the previous cohort but we were able to include electroconvulsive therapy factor and drug abuse rating scale. SV analysis identified 0 surrogate variables.

4. **GSE66937**. Suicide status (binary) was used as the main predictor. Models were adjusted for RNA integrity number and expression hybridization year batch. No other covariates were available. SV analysis identified 0 surrogate variables.
5. **GSE199536**. Suicide status was used as the main predictor. Models were adjusted for age, post-mortem interval, and tissue pH. All participants were male. SV analysis identified 5 SVs that were used.
6. **GSE92538 U133A**. Suicide status (binary) was used as the main predictor. Models were adjusted for sex, age, diagnosis, agonal factor (binary), post-mortem interval, tissue pH, ethnicity (race), and batch. Three SVs were used in the surrogate-adjusted analysis.
7. **GSE92538 U133 PLUS2.** We used the same covariates as in the previous cohort except for the agonal factor. It was not used as only one participant had value above 0. Two surrogate variables were used in the SV-adjusted analysis.
8. **GSE102556**. Suicide status (binary) was used as the main predictor. Models were adjusted for phenotype (MDD/control), sex, age, alcohol intake, medication intake, RNA integrity number, post-mortem interval, and tissue pH. Smoking and drug predictors were not used due to high missing counts and since they were not used in analysis in the initial publication. Medication and drug types were also not considered for the same reasons. Suicide status was derived from cause of death, and the originial variable was not used as it only explains variance in controls (accident and natural death). Five SVs were used for this dataset.
9. **GSE243356**. Suicide status (binary) was used as the only predictor. No other covariates were available in the phenotype data. Five SVs were used for this dataset.
10. **GSE248260**. Suicide status (binary) was used as the main predictor. We adjusted models for age, sex, post-mortem interval, tissue pH, RNA integrity number, and drug intake (binary). Diagnosis was not used as it was fully correlated with suicide status. Five SVs were used for this dataset.
11. **GSE247998**. Suicide status (binary) was used as the main predictor. Models were adjusted for age, sex, and diagnosis. Suicidal ideation group was not adjusted for as it was considered a part of main causal pathway. Five SVs were used for this dataset.
12. **GSE202537**. Suicide status (binary) was used as the main predictor. Models were adjusted for age, sex, BMI, disease state, tissue pH, post-mortem interval, ethnicity (race), and RNA integrity number. Corrected time of death and tissue storage time were not used as we did not consider these predictors as large confounders for suicide status and to enable better comparability with other cohorts. Manner of death was used to infer suicide status, and the originial variable was not included as it only explains variance in controls (accident and natural death). Library size was not used as normalization factors are calculated in the data analysis pipeline. Five SVs were used for this dataset.
13. **GSE101521**. Suicide status (binary) was used as the main predictor. We adjusted the models for age, sex, depression diagnosis, tissue pH, post-mortem interval, and RNA integrity number. Five SVs were used for this dataset.
14. **GSE144136**. Suicide status (binary) was used as the main predictor. Models were adjusted for estimated cell proportions (when bulking was performed for all cells from the same participant). Since sum of all cell proportions is linearly dependent from individual cell types and sums up to 1, we excluded proportions of microglia (as it was the smallest fraction of cells on average in all participants) to enable model convergence. No other suitable covariates were available in phenotypes. Five SVs were used for this dataset.

15. **GSE213982**. We used the same predictors as in the previous cohort. No other suitable covariates were available in phenotypes. Five SVs were used for this dataset.

1.9 Probe and transcript mapping to gene symbols

To enable comparison between studies, we needed to ensure that genes are named consistently across all datasets. First, probes in the array cohorts were mapped to gene symbols using provided annotation files from the latest versions from the manufacturers or GEO. If the annotation files provided insufficient/inconvenient data to map probes to genes, we used python connection to BioMart - Ensembl https://pypi.org/project/biomart/ to perform mapping of probe IDs to Ensembl gene IDs and symbols. This strategy was used in GSE208338, GSE66937, and GSE199536. Mapping of genes to biomart considered both approved HGNC symbols and alternative alias gene symbols. Mapping of RNAseq reads to Ensembl gene IDs was performed by *STAR* aligner directly. To ensure consistent naming of genes, we further harmonized names against identical reference dataset with approved symbols and synonyms. We used NIH NCBI Gene dataset Homo_sapiens.gene_info.gz from https://ftp.ncbi.nih.gov/gene/DATA/GENE_INFO/Mammalia/ as a reference.

1.10 Meta-analysis of differential expression changes

We performed meta-analysis for differential expression changes for all included genes if they passed both of the following selection criteria:

1. Gene was analyzed in at least five brain datasets. Estimates obtained from blood were not formally meta-analyzed but were used as side comparison
2. Gene was considered analyzed in the study if it had at least one probe that was uniquely annotated to its symbol. All probes that were annotated/aligned to several genes were discarded as ambiguous.

In some of the datasets several probes analyzed the same gene. In this case, we generated an aggregated differential expression measure for such genes. The effect size for such a gene was calculated as arithmetic mean of all individual log2FC for the gene in the study. Standard error (SE) for an aggregated measure was formulated as the maximal SE among all log2FC SEs observed for the gene in the study. Such a strategy provides the largest confidence interval (the lowest certainty) and represents conservative measure to represent gene expression measured by several probes.

$$Aggregated\ Log_2FC = \frac{\sum_{i=1}^{N} Log_2FC_i}{N}$$

$$Aggregated\ SE\ =\ max(S),\ where\ S\ =\ \{SE_1,\ SE_2,\ ...,SE_n\}$$

Here N denotes the number of probes corresponding to the same gene in a single study. The letter S denotes a set of standard errors for Log_2FC estimates for the same gene, and SE denotes standard error.

Since many potential strategies and factors could be considered when meta-analyzing individual studies, we focused on three most straightforward directions that were deemed reasonable based on the author's subjective judgment:

1. Meta-analysis of brain expression in all brain datasets (only one tissue per cohort). For GSE202537, we selected Nucleus accumbens (Nac) to represent the cohort as it had the largest number of available probes. In GSE102556 and GSE66937, we selected prefrontal cortex expression as it was the most common tissue in all cohorts. In total, 14 datasets were meta-analyzed.
2. Meta-analysis of brain expression in all cortical datasets (only one tissue per cohort). We included the following tissues: dorsolateral prefrontal cortex, orbitofrontal cortex, and temporal cortex. In the cortical meta-analysis, 11 datasets were meta-analyzed.
3. Meta-analysis of brain expression specifically in the dorsolateral prefrontal cortex. Here we included only 9 datasets corresponding to the prefrontal cortex (GSE66937 unspecified) and dorsolateral prefrontal cortex. Cohort GSE5389 was not included as it was specifically collected using orbitofrontal cortex samples. GSE66937 was included as authors assume primarily dorsolateral origin of these samples, even though it was not clearly indicated in the initial study.

Each of these specified meta-analyses was performed in three versions: *limma* estimates obtained without adjustment for covariates, with adjustment for covariates, or with adjustment of surrogate variables. Thus, nine meta-analyses were conducted in total. In the meta-analysis without covariates, the effects from suicide-related factors (such as depression/schizophrenia diagnosis) and suicide status are not separated and are considered as a part of same causal pathway. This analysis should capture genes that are associated not only to suicide but also to suicide-related psychiatric diagnoses and phenotypes. In the adjusted analyzes, the effects of co-morbidities are controlled for (depending on the dataset) and primarily suicide-specific changes should be identified. Surrogate variable-adjusted analysis, in turn, may provide a more standardized approach to consider variations within data.

The meta-analysis model was based on the random effects [44] approach implemented in the R package *metafor* with the function *rma.uni* [45]. As an input, we used *limma*-estimated log2FC (effect size) and its associated SE (vectors of the same length, n>=5). SE was squared and used as a sampling variance input (see *metafor* documentation). Weights for individual studies were calculated using the inverse-variance methods (*metafor* default). To enable model convergence for all genes, the amount of heterogeneity was set to Sidik-Jonkman estimator (also called model error variance estimator) [46, 47]. This estimator was selected due to simplicity (closed-form solution) and as we expected to observe high heterogeneity within studies and this estimator was shown to provide the most conservative estimate for heterogeneity with high positive bias for small heterogeneity and small bias if true heterogeneity is high [47, 48]. We considered the nominal significance of genes after meta-analysis. We also report FDR-corrected p-values calculated via R function *p.adjust* (method="fdr") for information purposes, though they were not further used. To select the most important genes, we focused on transcripts with absolute log2FC >= 0.2.

1.11 Sensitivity analyses and moderator analysis

We performed an alternative calculation for meta-analysis, using the restricted maximum likelihood estimator (REML) with parameters control=list(stepadj=0.5, maxiter=10000) to enable convergence for all transcripts and applied Hartung-Knapp-Sidik-Jonkman correction by setting test="knha" (as suggested by *metafor* documentation section "Tests and Confidence Intervals" in rma.uni {metafor}).

As an alternative strategy for the main meta-analysis, we also considered robust rank aggregation (RRA) meta-analysis. This method identifies significant genes based on relative positions of these genes in the set of ranked lists and how these positions are different from random lists [49]. The analysis method is implemented within the R package *RobustRankAggreg.* We added RRA calculation to every primary meta-analysis. To consider the directional nature of log2FC, RRA was performed separately for upregulated and downregulated lists from every initial cohort-level differential expression datasets. List ranking was based on p-values (lowest first). We used full ranked lists to perform RRA. In the results table, for every gene we present either RRA calculated from upregulated lists or from downregulated lists. Upregulated RRA was selected for genes with positive meta-estimated log2FC, and downregulated RRA was selected for genes with negative meta-estimated log2FC. If meta-estimated log2FC was not available (n cohorts<5), we selected direction based on majority from the number of cohorts where the gene is up- or down-regulated. If this number was equal, the positive RRA was selected. RRA p-values were adjusted with the FDR method.

We also performed meta-analyses with cohort-level moderators to investigate the potential effect of systemic differences across primary cohorts. Given that previous works suggest around 5-10 studies per single moderator [50], we only performed moderation analysis for genes detected in at least 10 datasets. We used several moderators in this analysis, including technology type array/RNAseq (binary), mean cohort PMI (numeric), percent of male participants in cohort (numeric), percent of depressed in cohort (numeric), percent of bipolar disorder in cohort (numeric), percent of schizophrenia in cohort (numeric). The platform was simplified to binary (array/RNAseq) to avoid including too many dummy variables to the models as data has at most 14 studies per gene. Other moderators were also considered and were not included for technical reasons. Chemistry to prepare RNA libraries was not possible to standardize across cohorts and it had single replicates for certain chemistry kits. Diagnoses for addiction disorders were either not provided in several datasets or used as exclusion. Other psychiatric diagnoses (non-addiction) were only provided by GSE66937. Tissues type was considered as moderator, however, we opted to use stratification as in the main meta-analysis. Sex ratio was considered instead of percent of male participants. However, some datasets had either only males or females, thus implying division by 0. Therefore, the percentage of male was selected. For some of the datasets, moderators were taken from the source publication since data at GEO did not contain such information. Below, we described data collection for moderators per study.

1. GSE208338 All moderators were calculated from GEO phenotype data.
2. GSE5388 All moderators were calculated from GEO phenotype data.
3. GSE5389 All moderators were calculated from GEO phenotype data.
4. GSE66937 GEO data did not contain data on PMI, sex, and diagnoses of participants. Related primary publication [8] reports data on a larger set of participants in tables 1 and 2. However, estimates obtained from such data would be inaccurate due to differences in samples. Therefore, GSE66937 was excluded from moderation analysis.
5. GSE199536 Mean PMI and percent of male were calculated using GEO phenotype data. Diagnosis information was available in the initial publication [9] Data S1 supplementary methods.
6. GSE92538_U133A and GSE92538_U133_PLUS2. All moderators were calculated from GEO phenotype data.
7. GSE102556 All moderators were calculated from GEO phenotype data.

8. GSE243356 Phenotype from GEO did not contain required data for moderators. The initial manuscript [16] reports that the sample median PMI was 15 hours, 34% of subjects in both groups were female, and all 29 suicide cases (47.5%) had MDD as their primary psychiatric diagnosis.
9. GSE248260 All moderators were calculated from GEO phenotype data.
10. GSE202537 All moderators were calculated from GEO phenotype data.
11. GSE101521 All moderators were calculated from GEO phenotype data.
12. GSE144136 and GSE213982. Phenotype from GEO did not contain required data for PMI. Mean PMI was calculated as weighted mean based on data from table 1 in initial publication [21]. This publication also states that all cases were affected by MDD, whereas controls did not have evidence for axis I disorders.

To perform moderation analysis we used R package metafor function rma.uni. Moderators were specified as formulas in the "mods" argument. Heterogeneity was estimated with the SJ estimator. Analyses were performed for initial DE data without covariates, with covariates, or with surrogate variables. We performed moderation analysis both in all brain tissues, as well as in the cortical subset. The prefrontal cortex subset contained less than 10 studies and was not performed due to a small study number.

1.12 Cell deconvolution analysis

Cell deconvolution meta-analysis was performed in several steps. First, representative signature matrices were constructed using the latest (as of Aug. 2025) docker version of CIBERSORTx [51]. Initial publication suggests that as low as 20 cells and 2-3 donors may be sufficient for a reliable signature matrix [51]. We tested multiple strategies for signature matrices, including sampling of all cells from 3 random male participants, sampling 10000 cells from 52 participants without balancing, sampling 30000 cells from 52 participants without balancing as well as trying to balance signature matrices by initial amount of included cell profiles. The resulting two best-performing matrices were used and described below. One matrix was derived from five males and five females sampled from GSE144136 (random seed 1234567) and GSE213982 (random seed 12345678), and covered 5 cases and 5 controls (referred as "sampled signature matrix"). The sampled signature matrix was based on 12173 cell profiles. We balanced sampled cells and included 2000 single cells for excitatory neurons and inhibitory neurons, while keeping all cell profiles for other cell types. This was done so other cell types have sufficient representation during the construction of the signature matrix. Random seeds were selected so initial participants include an equal number of males and females, and also cover an approximately equal number of cases and controls for both males and females. We sampled a total of 10 participants, so the remaining sample is sufficiently large to use in validation. Our tests showed that the number of samples in validation must be 3-4 times larger than the number of cell types to have better performance. This is supporting the initial CIBERSORTx methods [51] publication.

The second selected signature matrix, included grouping initial cell groups into three major categories ("neuronal", "glial", and "other"), and sampling cells from 10 males (seed 123456) and 10 females (seed 123456789) (each 5 cases and controls) (referred as "large sampled signature matrix simplified (LSSMS)"). Selected seeds were used to provide balanced numbers of males and females as well as cases with controls. We simplified astrocytes ("Ast"), microglia ("Mic"), oligodendrocytes ("Oli") and their precursor cells ("OPC") into the "glia" class. Exicatory neurons ("ExN") and inhibitory neurons ("InN") were simplified to "neuronal" class. Cells with mixed expression ("Mix") and endothelial cells ("End") were classified as "other". The simplification of cell types was made as reconstruction of more

specific subtypes was somewhat inaccurate even in validation dataset for the majority of cell classes in other tested signature matrices. This may indicate overfitting of the signature matrix or a large heterogeneity within cell types. The LSSMS matrix was designed to balance cell types in the input as cohorts GSE144136 and GSE213982 had the majority of cells belonging to the neuronal class. Therefore, we sampled 6000 excitatory and 6000 inhibitory neurons and used all cells of other types to construct LSSMS from ~ 27000 cells. A total of 20 participants were selected so there are enough remaining samples to perform validation. We picked 6000 cells for each type of neurons so a total of 12000 cells approximately matches the total count of 13601 cells for the glial subtype. The total number of single cell profiles in LSSMS was 27129. To ensure better compatibility of designed signature matrices with remaining bulk RNAseq cohorts, we filtered initial single cell profiles so they include only those genes that were detected in bulk RNAseq cohorts. Additionally, gene symbols were standardized across cohorts prior to construction of signature matrices so consistent gene symbols are used during deconvolution. Sampled signature matrix was built with fraction parameter set to 0 as suggested for scRNAseq signatures by CIBERSORTx manual. In LSSMS, we set fraction to 0.5 as we wanted to make this matrix generalisable while still having a large number of included cells to avoid generating a sparse signature matrix.

Validation samples for obtained signature matrices were constructed utilizing only cells from individuals that were not used during the construction of signature matrices. We both used separate single cells, but also avoided using the same participants in the validation sets. Performance was estimated as both spearman and pearson correlations of estimated and real cell proportions. Performance of high resolution mode was estimated as both spearman and pearson correlations between estimated expression and real pseudobulked expression of 5000 random genes. We used strict conditions with S-mode batch correction enabled during the validation of signature matrices. This was done to perform validation of deconvolution in exactly the same way as other bulk RNAseq cohorts.

Cell deconvolution of bulk RNAseq datasets and validation subsets was performed with the docker version of CIBERSORTx with enabled S-mode batch correction. Raw counts were used as inputs so the resulting expression values are compatible with the limma-voom pipeline. We inspected each run using mean variance trend plots so they follow expected trajectories. All transcripts mapping to the same gene symbol were discarded prior to deconvolution and matrices without duplicates were used. Imputed expression values filtered to exclude all genes that had more than 80% of identical expression estimations across all samples within individual cohorts. Then, these filtered expression values were further filtered by *filterByExpr* from *edgeR* and subject to the limma-voom DE pipeline that we used for bulk RNA-seq cohorts without covariates (described here in Section 1.8). In true single cell cohorts GSE144136 and GSE213982, we performed pseudobulking based on either initial cell types or simplified cell types and then used these counts for the same limma-voom DE pipeline.

Subsequently, obtained DE tables were further filtered to include genes that were nominally significant in GSE144136 or GSE213982. This restriction was made to ensure that meta-analysis only considers genes with some evidence of prior cell-specific differential expression. We did so to reduce the number of false positives as cell deconvolution may be inaccurate. The remaining genes were meta-analysed using the same primary random effects meta-analysis pipeline as described in section 2.8. We only analyzed genes that were successfully imputed and analyzable in at least 5 cohorts.

1.13 Gene enrichment analysis and classification

Gene set enrichment analysis was applied for all genes significant in meta-analysis (default approach). As a universe, we set all genes that were available for analysis. Mapping of genes to ENSEMBL gene ids was performed using NCBI gene dataset: https://ftp.ncbi.nih.gov/gene/DATA/GENE_INFO/Mammalia/. Enrichment analysis was performed for biological processes using the *clusterProfiler* R package [52].

Gene type classification was performed for significant genes with absolute log2FC >= 0.2. We used the OpenAI API with the GPT-5 model to perform classification. Reasoning effort was set to a low level. Each gene was classified one at a time. The model was given the ability to use "web_search_ preview" and instructed to use authoritative biological databases. We manually inspected results of classification and reclassified 5 genes entries.

The following prompt was applied:

You are given a human gene name presented as a gene symbol. You need to do the following:

1. Classify this gene using only one the most appropriate class (separated by ;):

'Receptor (non-immune)';

'Immune receptor';

'Ion channel/Transporter';

'Cell-Surface Ligand';

'Extracellular or Secreted molecule';

'Enzyme (non-kinase)';

'Transcription factor';

'Kinase';

'Structural protein';

'Non-coding RNA';

'Pseudogene'

2. You must only use scientific literature (papers) or scientific databases (such as UniProt, GeneCards, etc.) to perform classification

3. Give only one the most suitable class for a gene

4. Your output must be first an explanation of around 100 words then keyword "RESPONSE_CLASS" followed by the selected class name (spelled exactly as in the instruction 1.)

2. Descriptions for supplementary figures

All supplementary figures are presented as one single PDF file. The file is annotated so every page is a separate figure with its names corresponding to the name of the figure (i.e. Fig_S1.png, etc.)

Figure S1 Volcano plots of all meta-analyses

This figure shows venn diagrams comparing all three primary meta-analysis strategies across partitioning by tissue (all brain, cortical, dorsolateral prefrontal cortex). Numbers indicate nominally significant genes at $p<0.05$.

Figure S2 Correlations of meta log2FC across different tissue partitions

This figure shows pairwise scatter plots comparing alllog2FCs estimated in different tissue partitions for DEs without covariates. "Genes in both" show the number of genes analyzed in both tissue subsets (per plot) and presented as dots.

Figure S3 Meta-analysis network pruned by significance in RRA

This figure shows network visualization for nominally significant results obtained in all meta-analyses. Only the genes with meta-estimated absolute log2FC >= 0.1 and $P_{RRA} < 0.05$ are shown. Yellow nodes indicate meta-analyses without covariates. Blued nodes indicate meta-analyses with covariates. Light-blue nodes indicate analysis with surrogate variables. Red nodes show genes. Arrow edges indicate nominal significance for a gene in the corresponding analysis. Edge colors correspond to direction where red indicates upregulation in cases and green indicates downregulation. Edge thickness is proportional to the absolute values of log2FC. Orange dashed circle indicates genes that were significant in all three meta-analyses without covariates. Blue dashed circle indicates genes that were significant in meta-analysis both with and without covariates in all brain tissues.

Figure S4 Moderator effect correlations

This figure shows correlations of moderator's beta coefficients for percent of depression, percent of bipolar or percent of schizophrenia from meta-regression models. Green scatterplot shows correlations of beta for % of depression in a cohort vs beta for % of bipolar disorder in a cohort in all brain subset for every gene included in this meta-regression. Brick-red scatterplot shows similar correlation but in the cortical meta-regression. Blue scatterplot shows correlations of beta for % of depression in a cohort vs beta for % of schizophrenia in a

cohort in cortical subset for every gene included in this meta-regression. All figures consider meta-regressions conducted on DEs without covariates.

Figure S5 Blood-brain log2FC correlation

This plot shows correlation of log2FC obtained from meta-analysis without covariates with all brain tissues against average log2FC obtained from the blood cohort GSE247998. Only genes that passed QC and were analyzed in both analyses were plotted

Figure S6 Performance of LSSMS matrix to reconstruct validation fractions

This figure shows reconstruction of cell proportions for pseudobulked validation profiles from GSE144136 and GSE213982 using LSSMS. These plots show reconstructions for broad glial type, broad neuronal type, and other cell type. X-axis show real proportions estimated by counting real cell profiles in pseudobulked validation samples. Y-axis shows predicted proportions for corresponding validation samples. The blue line indicates fitted linear regression. There are no single cells or participants that overlap between data used to construct LSSMS signature matrix and validation samples for this matrix.

Figure S7 Performance of sampled signature matrix to reconstruct validation fractions

This figure shows reconstruction of cell proportions for pseudobulked validation profiles from GSE144136 and GSE213982 using sampled signature matrix. These plots show reconstructions for all cell types as were defined in the initial single cell cohorts. X-axis show real proportions estimated by counting real cell profiles in pseudobulked validation samples. Y-axis shows predicted proportions for corresponding validation samples. The blue line indicates fitted linear regression. There are no single cells or participants that overlap between data used to construct the sampled signature matrix and validation samples for this matrix.

Figure S8 Performance of high resolution mode to reconstruct cell-type-specific expression with LSSMS

This figure shows reconstruction of cell-specific expression for pseudobulked validation profiles from GSE144136 and GSE213982 using LSSMS. These plots show reconstructions for broad glial type, broad neuronal type, and other cell type. X-axis show real transcript counts as estimated by pseudobulking real cell counts into simplified cell-type specific expressions in validation samples. Y-axis shows estimated cell-type-specific counts for corresponding validation samples. The blue line indicates fitted linear regression. We sampled 5000 random genes to construct each scatterplot. There are no single cells or participants that overlap between data used to construct LSSMS signature matrix and validation samples for this matrix.

Figure S9 Performance of high resolution mode to reconstruct cell-type-specific expression with sampled signature matrix

This figure shows reconstruction of cell-specific expression for pseudobulked validation profiles from GSE144136 and GSE213982 using sampled signature matrix. These plots show reconstructions for broad glial type, broad neuronal type, and other cell type. X-axis show real transcript counts as estimated by pseudobulking real cell counts into cell-type specific expressions as defined in the primary cell cohorts in validation samples. Y-axis shows

estimated cell-type-specific counts for corresponding validation samples. The blue line indicates fitted linear regression. We sampled 5000 random genes to construct each scatterplot. There are no single cells or participants that overlap between data used to construct the sampled signature matrix and validation samples for this matrix.

Figure S10 GO Biological process enrichment for DLPFC analysis (without covariates)

This figure shows top enriched biological processes for DLPC analysis without covariates. P-values were adjusted with the false discovery rate method. Min category size was set to 10 and enrichment was performed with the R package *clusterProfiler*.

Figure S11 GO Cellular component enrichment for DLPFC analysis (without covariates)

This figure shows top enriched cellular components for DLPFC analysis without covariates. P-values were adjusted with the false discovery rate method. Min category size was set to 10 and enrichment was performed with the R package *clusterProfiler*.

3. Descriptions for supplementary materials

Data S2 This table provides demographic information for the PSY cohort separated based on SUAS risk score. Numeric values are shown as mean ± standard deviation, minimal and maximal values. Categorical values are shown as counts per group and corresponding percentage within a group.

Data S3 This excel file provides several tables individually showing demographic data of individual cohorts/datasets from GEO. Each sheet is named in connection to the dataset. Every cohort was separated into two groups based on suicide status. If a cohort had data on several tissues, each of the tissues was provided with demographic annotation separately. Numeric values are shown as mean ± standard deviation, minimal and maximal values. Categorical values are shown as counts per group and corresponding percentage within a group.

Data S4 This table provides results for differential proteomic analysis in the PSY cohort. Columns indicate the following: logFC, estimate of the log2-fold-change; AveExpr, average log2-expression for the probe over all arrays and channels; t, moderated t-statistic; P.Value, raw p-value; adj.P.Value, adjusted p-value or q-value; B, log-odds that the gene is differentially expressed; Gene, gene symbol.

Data S5 This file shows statistics for individual differential expression analyses without covariates per cohort. Columns denote the following: tissue - tissue of the samples (could be several in one cohort), total_associations - total number of models tested (per tissue), unique_gene_symbols_p_lt_0_05 - number of unique gene symbols with p<0.05 (per tissue), unique_IDs_p_lt_0_05 - number of unique probe IDs with p<=0.05 (per tissue) (for RNA seq ID usually match gene symbols with few exceptions), unique_gene_symbols_adj_p_lt_0_05 - number of unique gene symbols with FDR-adjusted p<=0.05 (per tissue), unique_IDs_adj_p_lt_0_05 - number of unique probe IDs with FDR-adjusted p<=0.05 (per tissue), mean_abs_log2FC - average absolute effect size for a transcript in all models, mean_abs_log2FC_nomin_signif - average absolute effect size for a transcript in all models where nominal p<=0.05.

Data S6 This file shows statistics for individual differential expression analyses with the use of covariates per cohort. Columns denote the same as in Data S5.

Data S7 This file shows statistics for individual differential expression analyses with the use of surrogate variables per cohort. Columns denote the same as in Data S5.

Data S8 This excel file provides results for the meta-analysis using Sidik-Jonkman estimator for heterogeneity. Each sheet corresponds to individual meta-analysis. Columns indicate the following: Gene, gene symbol; detected cohorts, number of cohorts where gene was analyzed (after QC); cohorts up, number of cohorts where the gene was upregulated (mean higher in cases), number of cohorts where the gene was downregulated (mean lower in cases); commentary, a commentary if meta-analysis was conducted; meta_LFc, meta-estimated log2FC; meta_se, meta-estimated standard error; meta_pval, meta-estimated p-value (raw); tau2, tau2 estimate of heterogeneity; I2, I2 estimate of heterogeneity; H2, H2 statistic; Q, test statistic of the test for (residual) heterogeneity; Q.p, p-value for Q; mean_blood_lfc, average log2FC in blood for the same gene, blood_dir, probe direction of change in blood (with respect to cases); blood_signif, nominal significance of blood changes; matching_with_blood, a binary indicating directional match/mismatch with brain; P_RRA,

p-values for robust rank aggregation analysis per gene (either RRA on upregulated or downregulated lists was selected); FDR_RRA p-values for robust rank aggregation analysis per gene corrected by the false discovery rate method; N_cohorts_RRA, number of cohorts used in RRA analysis (either includes upregulated lists or downregulated lists).

Data S9 This folder archive provides all Forest plots for all significant genes in all nine primary meta-analyzes. Forest plots were created with the R package metafor

Data S10 This folder provides volcano plots in all nine meta-analyzes. X-axis corresponds to meta-estimated log2FC, whereas the Y-axis indicates meta -log10(p-value). Blue line and red line indicate p-values 0.05 and 0.001, respectively. Gray lines indicate log2FC threshold of 0.2

Data S11 This folder provides funnel plots for every nominally significant gene in all primary meta-analyses. The X-axis corresponds to log2FC from primary differential expressions, whereas the Y-axis indicates the corresponding standard error of these estimates. Dotted line shows average effect.

Data S12 This table shows estimated heterogeneity (I2 and tau2) across non-moderated meta-analyses. For each analysis and metric, minimal, median, and maximal values are provided.

Data S13 This text document shows pairwise comparisons of meta-analyses results with respect to DE adjustment strategies as well as tissue subsetting.

Data S14 This excel file provides results for the meta-analysis using REML estimator for heterogeneity and Hartung-Knapp-Sidik-Jonkman correction for inference. Each sheet corresponds to individual meta-analysis. Columns indicate the following: Gene, gene symbol; detected cohorts, number of cohorts where gene was analyzed (after QC); cohorts up, number of cohorts where the gene was upregulated (mean higher in cases), number of cohorts where the gene was downregulated (mean lower in cases); commentary, a commentary if meta-analysis was conducted; meta_LFc, meta-estimated log2FC; meta_se, meta-estimated standard error; meta_pval, meta-estimated p-value (raw); tau2, tau2 estimate of heterogeneity; I2, I2 estimate of heterogeneity; H2, H2 statistic; Q, test statistic of the test for (residual) heterogeneity; Q.p, p-value for Q; mean_blood_lfc, average log2FC in blood for the same gene, blood_dir, probe direction of change in blood (with respect to cases); blood_signif, nominal significance of blood changes; matching_with_blood, a binary indicating directional match/mismatch with brain; P_RRA, p-values for robust rank aggregation analysis per gene (either RRA on upregulated or downregulated lists was selected); FDR_RRA p-values for robust rank aggregation analysis per gene corrected by the false discovery rate method; N_cohorts_RRA, number of cohorts used in RRA analysis (either includes upregulated lists or downregulated lists).

Data S15 This text file compares results from random effects meta-analyses with corresponding results from robust rank aggregation meta-analyses.

Data S16 This document shows results for moderator analysis. Cohort moderators sheet provides a list of cohort-level moderators that were used or considered in the analysis. Red cells indicate unusable values. Yellow cells indicate approximate values that were derived from paper publications rather than phenotype data from GEO. Subsequent sheets provide results for meta-regression in all brain tissue subset and in cortical subset with respect to not

using covariates, using phenotype covariates, or using surrogate variables. Columns indicate the following: gene, gene symbol; init_meta_logFC, initial meta-estimated log2FC from corresponding RE meta-analysis without moderation; init_meta_pval, meta-estimated p-value from corresponding RE meta-analysis without moderation; detected cohorts, number of cohorts where gene was analyzed (after QC); cohorts up, number of cohorts where the gene was upregulated (mean higher in cases), number of cohorts where the gene was downregulated (mean lower in cases); commentary, a commentary if meta-analysis was conducted; beta_intrcpt, beta-coefficient from meta-regression for the intercept; beta_Platform_binaryRNAseq, beta-coefficient from meta-regression for the binary moderator of platform (array was default); beta_Mean_PMI_hours, beta-coefficient from meta-regression for the numeric moderator mean PMI in a cohort; beta_Percent_male, beta-coefficient from meta-regression for the numeric moderator percent of male participants in a cohort; beta_Percent_Depr & beta_Percent_BD & beta_Percent_SCZ, beta-coefficients from meta-regression for corresponding numeric moderators of percentage of participants with a specified psychiatric diagnosis in a cohort; pval_<coefficient_name>, associated p-values from meta-regressions for abovementioned coefficients, tau2, tau2 estimate of heterogeneity; I2, I2 estimate of heterogeneity; H2, H2 statistic; Q, test statistic of the test for (residual) heterogeneity; Q.p, p-value for Q; QM, test statistic of the omnibus test of moderators; QMp, p-value for the omnibus test of moderators; R2, amount of heterogeneity that could be explained by meta-regression model.

Data S17 This text file provides summary stats of each moderator analysis calculated from tables in Data S16.. Values in parenthesis next to the gene symbols show corresponding beta coefficients rounded to two digits.

Data S18 This file provides statistics regarding matching of genes from each meta-analysis in the brain with a blood cohort GSE247998. We calculated the percentage of observed direction of changes, whether it reached nominal statistical significance, percentage of matching directions for analyzed genes, as well as extracted gene names that matched and mis-matched with blood.

Data S19. This excel file provides results for the cell deconvoluted meta-analysis using Sidik-Jonkman estimator for heterogeneity. Each sheet corresponds to individual cell-type meta-analysis (ExN, Neuronal, Glial). Columns indicate the following: Gene, gene symbol; cell group, deconvoluted cell group; detected cohorts, number of cohorts where gene was analyzed (after QC); cohorts up, number of cohorts where the gene was upregulated (mean higher in cases), number of cohorts where the gene was downregulated (mean lower in cases); commentary, a commentary if meta-analysis was conducted; meta_LFc, meta-estimated log2FC; meta_se, meta-estimated standard error; meta_pval, meta-estimated p-value (raw); tau2, tau2 estimate of heterogeneity; I2, I2 estimate of heterogeneity; H2, H2 statistic; Q, test statistic of the test for (residual) heterogeneity; Q.p, p-value for Q; supported analyses, text field showing names of associated primary bulk meta-analyses where associated gene was also nominally significant (regardless of direction). Subsequent sheets show results for KEGG pathway enrichments of the associated gene lists for the KEGG pathway mapped genes. P-values were adjusted with the false discovery rate method. Min category size was set to 10 and enrichment was performed with the R package *clusterProfiler.*

Data S20 This excel file provides enrichment for biological processes for differentially expressed genes that were mapped to the GO biological process in every meta-analysis. Each meta-analysis strategy is shown in individual sheets. P-values were adjusted with the false

discovery rate method. Min category size was set to 10 and enrichment was performed with the R package *clusterProfiler.*

4. References

1. Barrett T, Wilhite SE, Ledoux P, Evangelista C, Kim IF, Tomashevsky M, et al. NCBI GEO: archive for functional genomics data sets--update. Nucleic Acids Res. 2013;41:D991-995.
2. Stanley B, Träskman-Bendz L, Stanley M. The suicide assessment scale: a scale evaluating change in suicidal behavior. Psychopharmacol Bull. 1986;22:200–205.
3. Assarsson E, Lundberg M, Holmquist G, Björkesten J, Thorsen SB, Ekman D, et al. Homogenous 96-plex PEA immunoassay exhibiting high sensitivity, specificity, and excellent scalability. PLoS One. 2014;9:e95192.
4. Scarr E, Udawela M, Thomas EA, Dean B. Changed gene expression in subjects with schizophrenia and low cortical muscarinic M1 receptors predicts disrupted upstream pathways interacting with that receptor. Mol Psychiatry. 2018;23:295–303.
5. Worf K, Matosin N, Gerstner N, Fröhlich AS, Koller AC, Degenhardt F, et al. Exon-variant interplay and multi-modal evidence identify endocrine dysregulation in severe psychiatric disorders impacting excitatory neurons. Transl Psychiatry. 2025;15:153.
6. Worf K, Matosin N, Gerstner N, Fröhlich AS, Koller AC, Degenhardt F, et al. Variant-risk-exon interplay impacts circadian rhythm and dopamine signaling pathway in severe psychiatric disorders. 2022.
7. Ryan MM, Lockstone HE, Huffaker SJ, Wayland MT, Webster MJ, Bahn S. Gene expression analysis of bipolar disorder reveals downregulation of the ubiquitin cycle and alterations in synaptic genes. Mol Psychiatry. 2006;11:965–978.
8. Glavan D, Gheorman V, Gresita A, Hermann DM, Udristoiu I, Popa-Wagner A. Identification of transcriptome alterations in the prefrontal cortex, hippocampus, amygdala and hippocampus of suicide victims. Sci Rep. 2021;11:18853.
9. Kim HJ, Yoo H, Kim JY, Yang SH, Lee HW, Lee H-J, et al. Postmortem gene expression profiles in the habenulae of suicides: implication of endothelial dysfunction in the neurovascular system. Mol Brain. 2022;15:48.
10. Hagenauer MH, Schulmann A, Li JZ, Vawter MP, Walsh DM, Thompson RC, et al. Inference of cell type content from human brain transcriptomic datasets illuminates the effects of age, manner of death, dissection, and psychiatric diagnosis. PLoS One. 2018;13:e0200003.
11. Li JZ, Bunney BG, Meng F, Hagenauer MH, Walsh DM, Vawter MP, et al. Circadian patterns of gene expression in the human brain and disruption in major depressive disorder. Proc Natl Acad Sci U S A. 2013;110:9950–9955.
12. Evans SJ, Choudary PV, Vawter MP, Li J, Meador-Woodruff JH, Lopez JF, et al. DNA microarray analysis of functionally discrete human brain regions reveals divergent transcriptional profiles. Neurobiol Dis. 2003;14:240–250.
13. Li JZ, Vawter MP, Walsh DM, Tomita H, Evans SJ, Choudary PV, et al. Systematic changes in gene expression in postmortem human brains associated with tissue pH and terminal medical conditions. Hum Mol Genet. 2004;13:609–616.
14. Tomita H, Vawter MP, Walsh DM, Evans SJ, Choudary PV, Li J, et al. Effect of agonal and postmortem factors on gene expression profile: quality control in microarray analyses of postmortem human brain. Biol Psychiatry. 2004;55:346–352.
15. Labonté B, Engmann O, Purushothaman I, Menard C, Wang J, Tan C, et al. Sex-specific transcriptional signatures in human depression. Nat Med. 2017;23:1102–1111.
16. Sha Q, Fu Z, Escobar Galvis ML, Madaj Z, Underwood MD, Steiner JA, et al. Integrative transcriptome- and DNA methylation analysis of brain tissue from the temporal pole in

suicide decedents and their controls. Mol Psychiatry. 2024;29:134–145.
17. Sun S, Liu Q, Wang Z, Huang Y-Y, Sublette ME, Dwork AJ, et al. Brain and blood transcriptome profiles delineate common genetic pathways across suicidal ideation and suicide. Mol Psychiatry. 2024;29:1417–1426.
18. Ketchesin KD, Zong W, Hildebrand MA, Scott MR, Seney ML, Cahill KM, et al. Diurnal Alterations in Gene Expression Across Striatal Subregions in Psychosis. Biol Psychiatry. 2023;93:137–148.
19. Pantazatos SP, Huang Y-Y, Rosoklija GB, Dwork AJ, Arango V, Mann JJ. Whole-transcriptome brain expression and exon-usage profiling in major depression and suicide: evidence for altered glial, endothelial and ATPase activity. Mol Psychiatry. 2017;22:760–773.
20. Nagy C, Maitra M, Tanti A, Suderman M, Théroux J-F, Davoli MA, et al. Single-nucleus transcriptomics of the prefrontal cortex in major depressive disorder implicates oligodendrocyte precursor cells and excitatory neurons. Nat Neurosci. 2020;23:771–781.
21. Maitra M, Mitsuhashi H, Rahimian R, Chawla A, Yang J, Fiori LM, et al. Cell type specific transcriptomic differences in depression show similar patterns between males and females but implicate distinct cell types and genes. Nat Commun. 2023;14:2912.
22. Ritchie ME, Phipson B, Wu D, Hu Y, Law CW, Shi W, et al. limma powers differential expression analyses for RNA-sequencing and microarray studies. Nucleic Acids Res. 2015;43:e47.
23. Guldvik IJ, Ramberg H, Kristensen G, Røder A, Mills IG, Lilleby W, et al. Systemic interrogation of immune-oncology-related proteins in patients with locally advanced prostate cancer undergoing androgen deprivation and intensity-modulated radiotherapy. World J Urol. 2024;42:95.
24. Wang X, Yip KC, He A, Tang J, Liu S, Yan R, et al. Plasma Olink Proteomics Identifies CCL20 as a Novel Predictive and Diagnostic Inflammatory Marker for Preeclampsia. J Proteome Res. 2022;21:2998–3006.
25. Al-Nesf MAY, Abdesselem HB, Bensmail I, Ibrahim S, Saeed WAH, Mohammed SSI, et al. Prognostic tools and candidate drugs based on plasma proteomics of patients with severe COVID-19 complications. Nat Commun. 2022;13:946.
26. Farutin V, Kurtagic E, Pradines JR, Capila I, Mayes MD, Wu M, et al. Multiomic study of skin, peripheral blood, and serum: is serum proteome a reflection of disease process at the end-organ level in systemic sclerosis? Arthritis Res Ther. 2021;23:259.
27. Irizarry RA, Hobbs B, Collin F, Beazer-Barclay YD, Antonellis KJ, Scherf U, et al. Exploration, normalization, and summaries of high density oligonucleotide array probe level data. Biostatistics. 2003;4:249–264.
28. Leek JT, Johnson WE, Parker HS, Jaffe AE, Storey JD. The sva package for removing batch effects and other unwanted variation in high-throughput experiments. Bioinformatics. 2012;28:882–883.
29. Davis S, Meltzer PS. GEOquery: a bridge between the Gene Expression Omnibus (GEO) and BioConductor. Bioinformatics. 2007;23:1846–1847.
30. Gautier L, Cope L, Bolstad BM, Irizarry RA. affy--analysis of Affymetrix GeneChip data at the probe level. Bioinformatics. 2004;20:307–315.
31. Carvalho BS, Irizarry RA. A framework for oligonucleotide microarray preprocessing. Bioinformatics. 2010;26:2363–2367.
32. Andrews S. (2010). FastQC: a quality control tool for high throughput sequence data. Available online at: http://www.bioinformatics.babraham.ac.uk/projects/fastqc.
33. Chen S, Zhou Y, Chen Y, Gu J. fastp: an ultra-fast all-in-one FASTQ preprocessor. Bioinformatics. 2018;34:i884–i890.
34. Liao Y, Shi W. Read trimming is not required for mapping and quantification of RNA-seq

reads at the gene level. NAR Genom Bioinform. 2020;2:lqaa068.
35. Dobin A, Davis CA, Schlesinger F, Drenkow J, Zaleski C, Jha S, et al. STAR: ultrafast universal RNA-seq aligner. Bioinformatics. 2013;29:15–21.
36. Hao Y, Stuart T, Kowalski MH, Choudhary S, Hoffman P, Hartman A, et al. Dictionary learning for integrative, multimodal and scalable single-cell analysis. Nat Biotechnol. 2024;42:293–304.
37. Chen Y, Chen L, Lun ATL, Baldoni PL, Smyth GK. edgeR 4.0: powerful differential analysis of sequencing data with expanded functionality and improved support for small counts and larger datasets. 2024.
38. Chen Y, Lun ATL, Smyth GK. From reads to genes to pathways: differential expression analysis of RNA-Seq experiments using Rsubread and the edgeR quasi-likelihood pipeline. F1000Res. 2016;5:1438.
39. Law CW, Chen Y, Shi W, Smyth GK. voom: Precision weights unlock linear model analysis tools for RNA-seq read counts. Genome Biol. 2014;15:R29.
40. Steegen S, Tuerlinckx F, Gelman A, Vanpaemel W. Increasing Transparency Through a Multiverse Analysis. Perspect Psychol Sci. 2016;11:702–712.
41. Gelman A, Loken E. The garden of forking paths: Why multiple comparisons can be a problem, even when there is no “fishing expedition” or “p-hacking” and the research hypothesis was posited ahead of time*. 2013.
42. Simmons JP, Nelson LD, Simonsohn U. False-positive psychology: undisclosed flexibility in data collection and analysis allows presenting anything as significant. Psychol Sci. 2011;22:1359–1366.
43. Leek JT. Asymptotic conditional singular value decomposition for high-dimensional genomic data. Biometrics. 2011;67:344–352.
44. Dettori JR, Norvell DC, Chapman JR. Fixed-Effect vs Random-Effects Models for Meta-Analysis: 3 Points to Consider. Global Spine J. 2022;12:1624–1626.
45. Viechtbauer W. Conducting Meta-Analyses in *R* with the **metafor** Package. J Stat Soft. 2010;36.
46. Sidik K, Jonkman JN. Simple Heterogeneity Variance Estimation for Meta-Analysis. Journal of the Royal Statistical Society Series C: Applied Statistics. 2005;54:367–384.
47. Sidik K, Jonkman JN. A comparison of heterogeneity variance estimators in combining results of studies. Stat Med. 2007;26:1964–1981.
48. Langan D, Higgins JPT, Jackson D, Bowden J, Veroniki AA, Kontopantelis E, et al. A comparison of heterogeneity variance estimators in simulated random-effects meta-analyses. Res Synth Methods. 2019;10:83–98.
49. Kolde R, Laur S, Adler P, Vilo J. Robust rank aggregation for gene list integration and meta-analysis. Bioinformatics. 2012;28:573–580.
50. van Houwelingen HC, Arends LR, Stijnen T. Advanced methods in meta-analysis: multivariate approach and meta-regression. Stat Med. 2002;21:589–624.
51. Steen CB, Liu CL, Alizadeh AA, Newman AM. Profiling Cell Type Abundance and Expression in Bulk Tissues with CIBERSORTx. Methods Mol Biol. 2020;2117:135–157.
52. Wu T, Hu E, Xu S, Chen M, Guo P, Dai Z, et al. clusterProfiler 4.0: A universal enrichment tool for interpreting omics data. Innovation (Camb). 2021;2:100141.
