# Supplementary material for: Identification of suicide brain transcriptomic signatures using meta-analysis of multiple cohorts
Source: Transl Psychiatry. 2026 Mar 31;16:222. doi: 10.1038/s41398-026-03978-8 (PMC13039836; doi:10.1038/s41398-026-03978-8)

Fig_S1_Venn_Meta_comparison.png

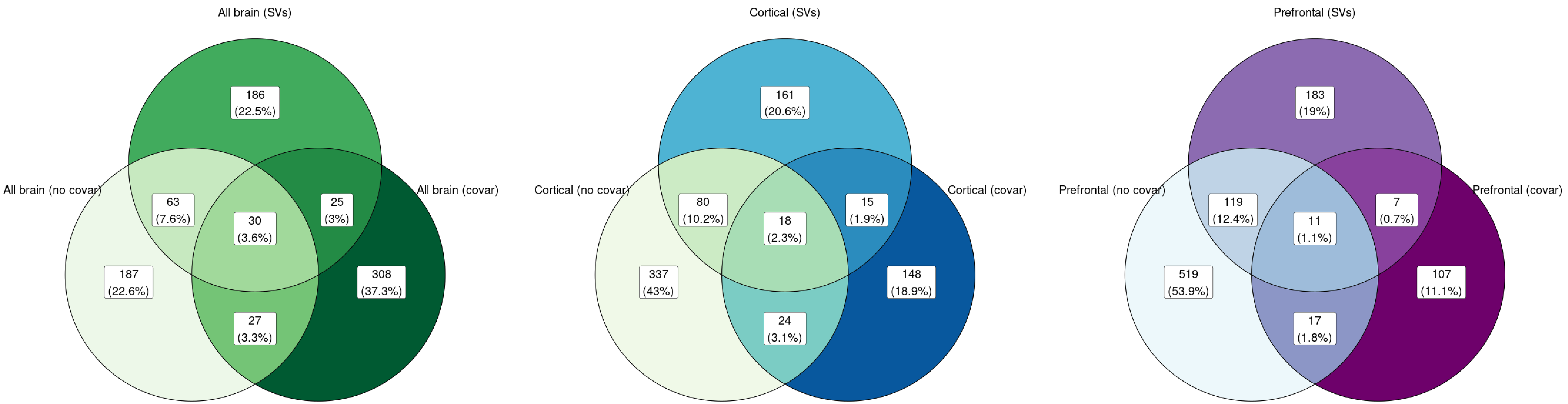

Fig_S2_Meta_effect_correlations_all.png

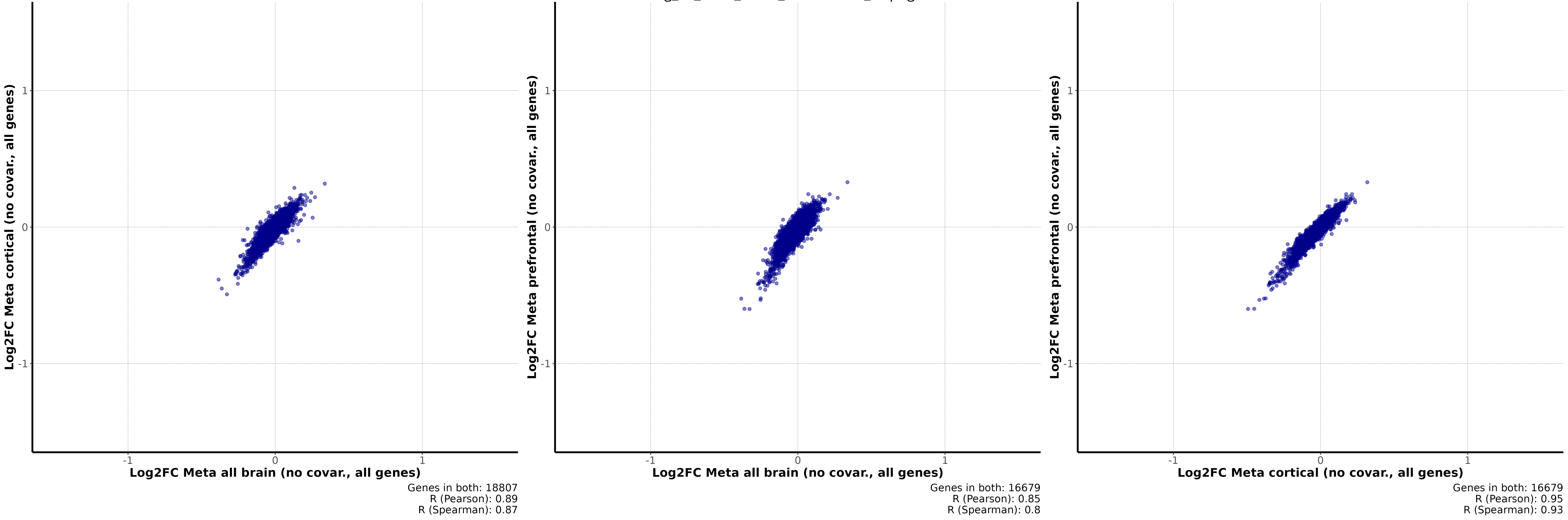

Fig_S3_Meta_net_RRA_RE.png

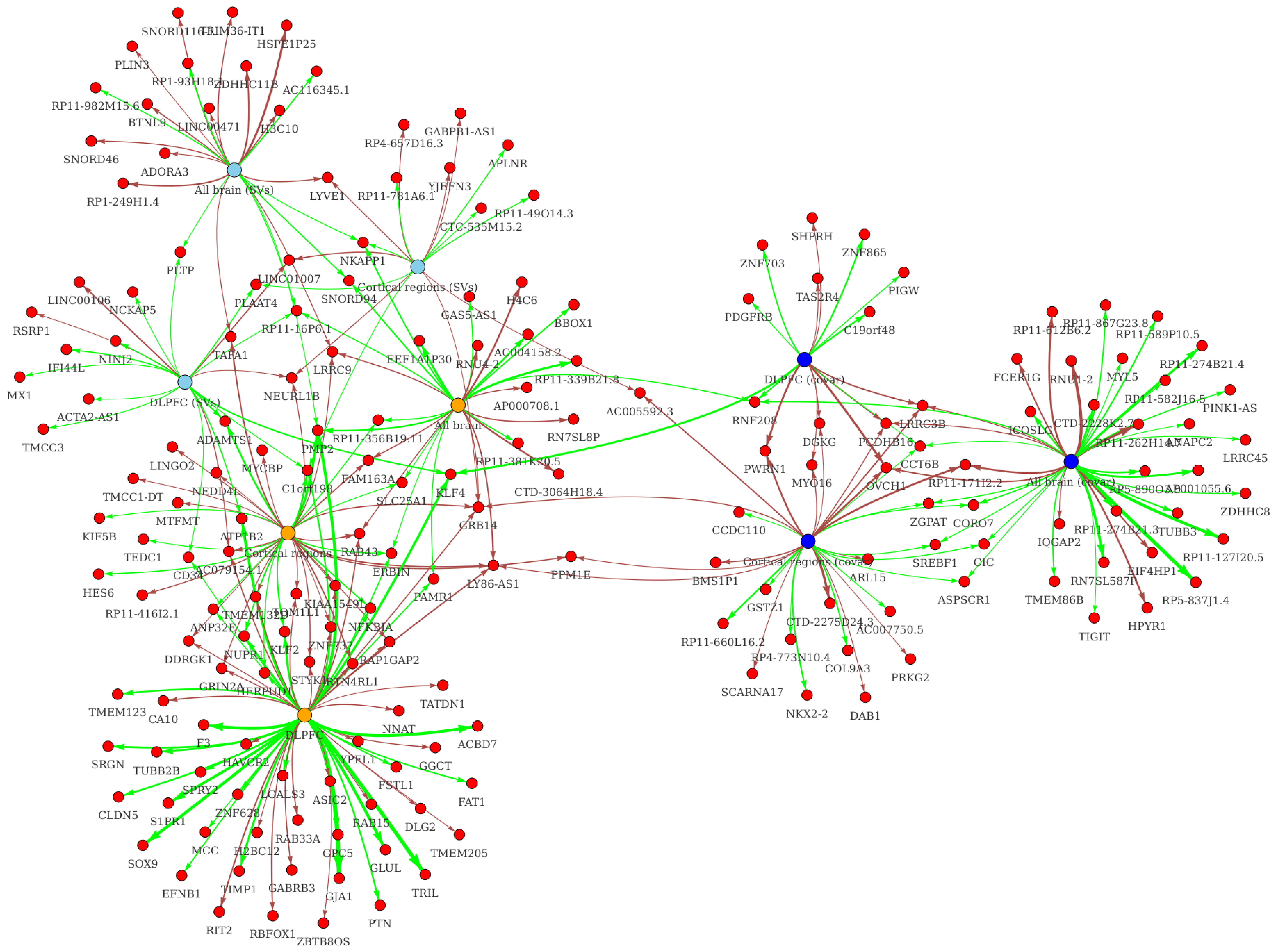

Fig_S4_Moderator_correlations.png

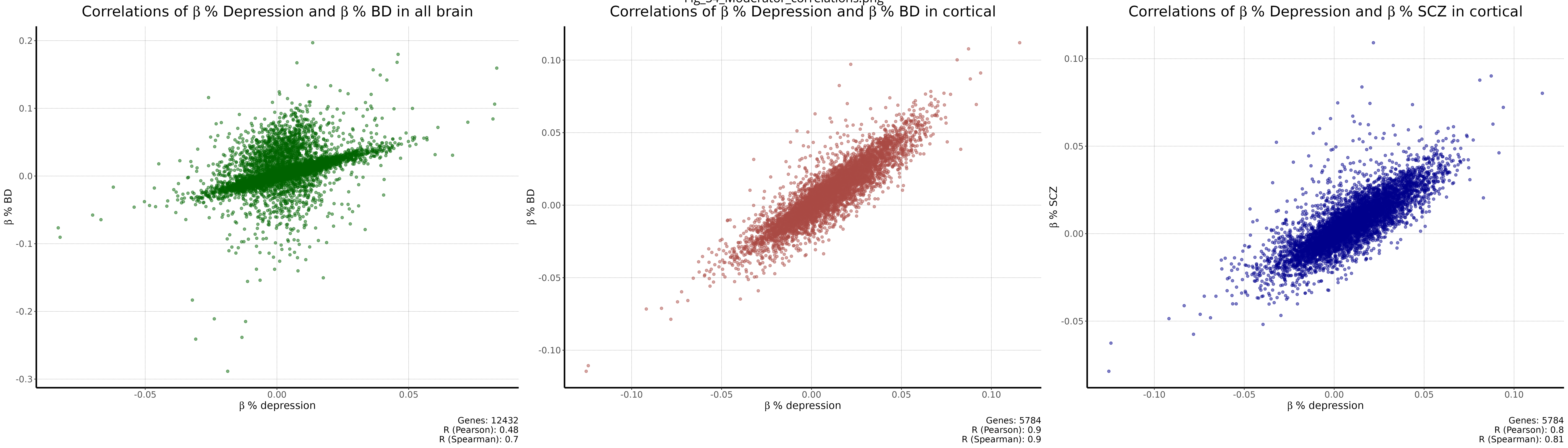

Fig_S5_Blood_brain_cor.png

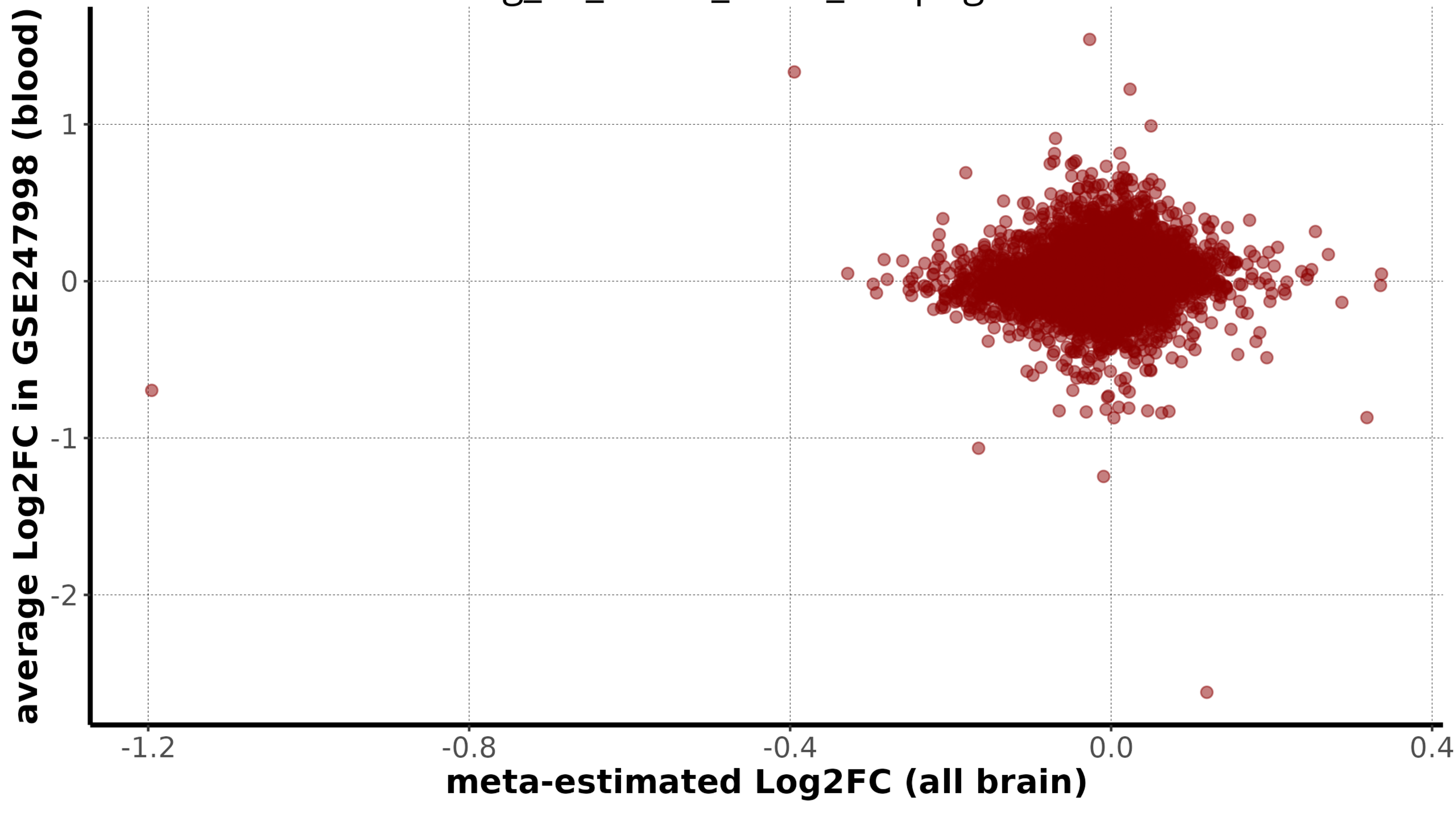

Fig_S6_LSSMS_fractions_validation.png

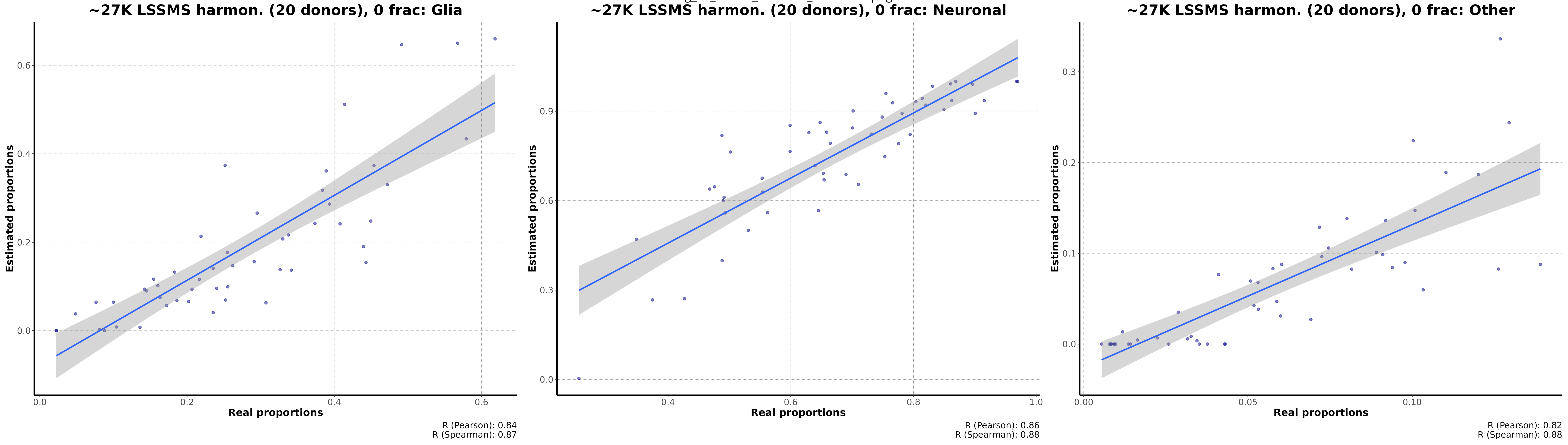

Fig_S7_Sampled_signature_fractions_val.png

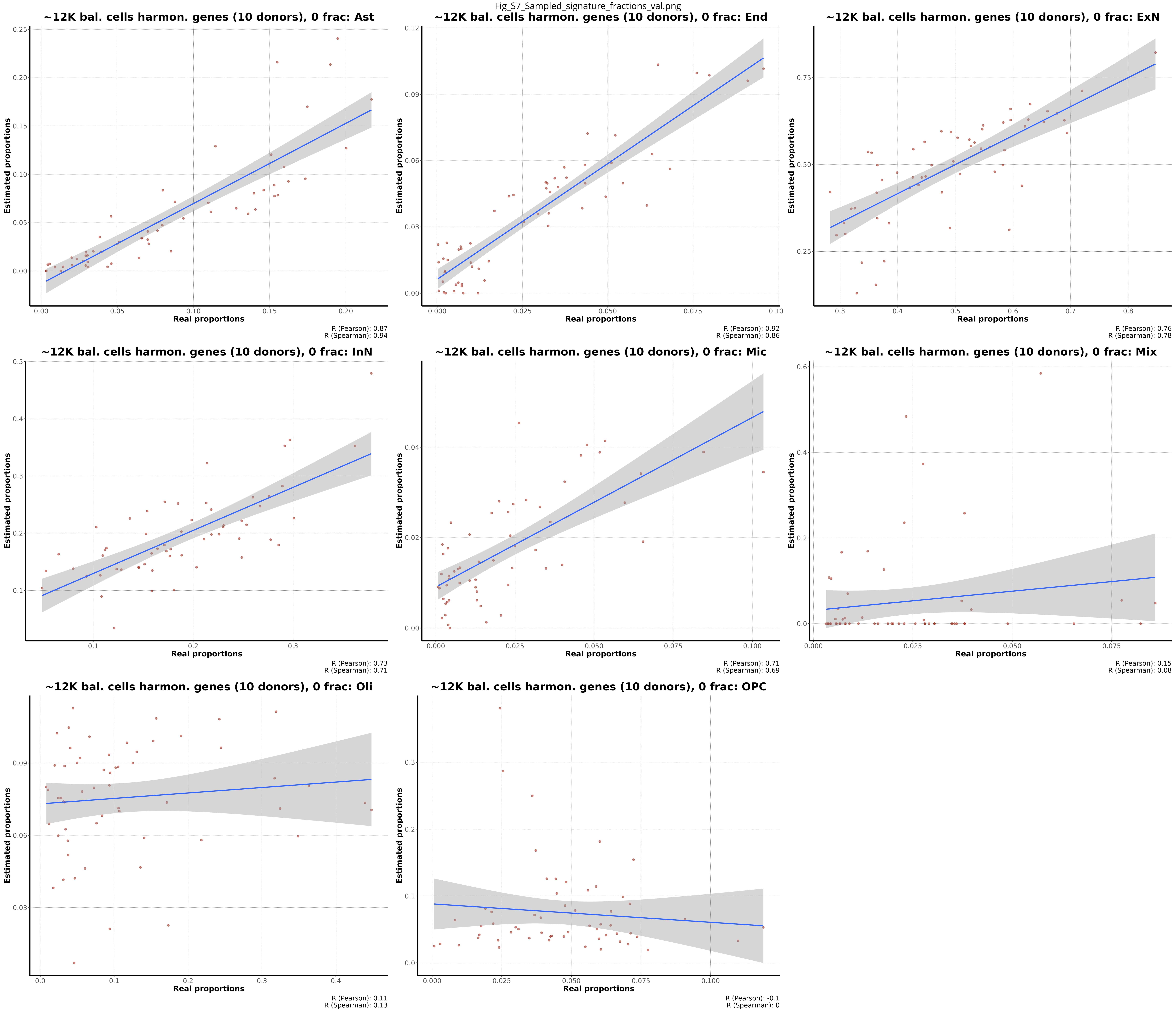

Fig_S8_LSSMS_highres_validation.png

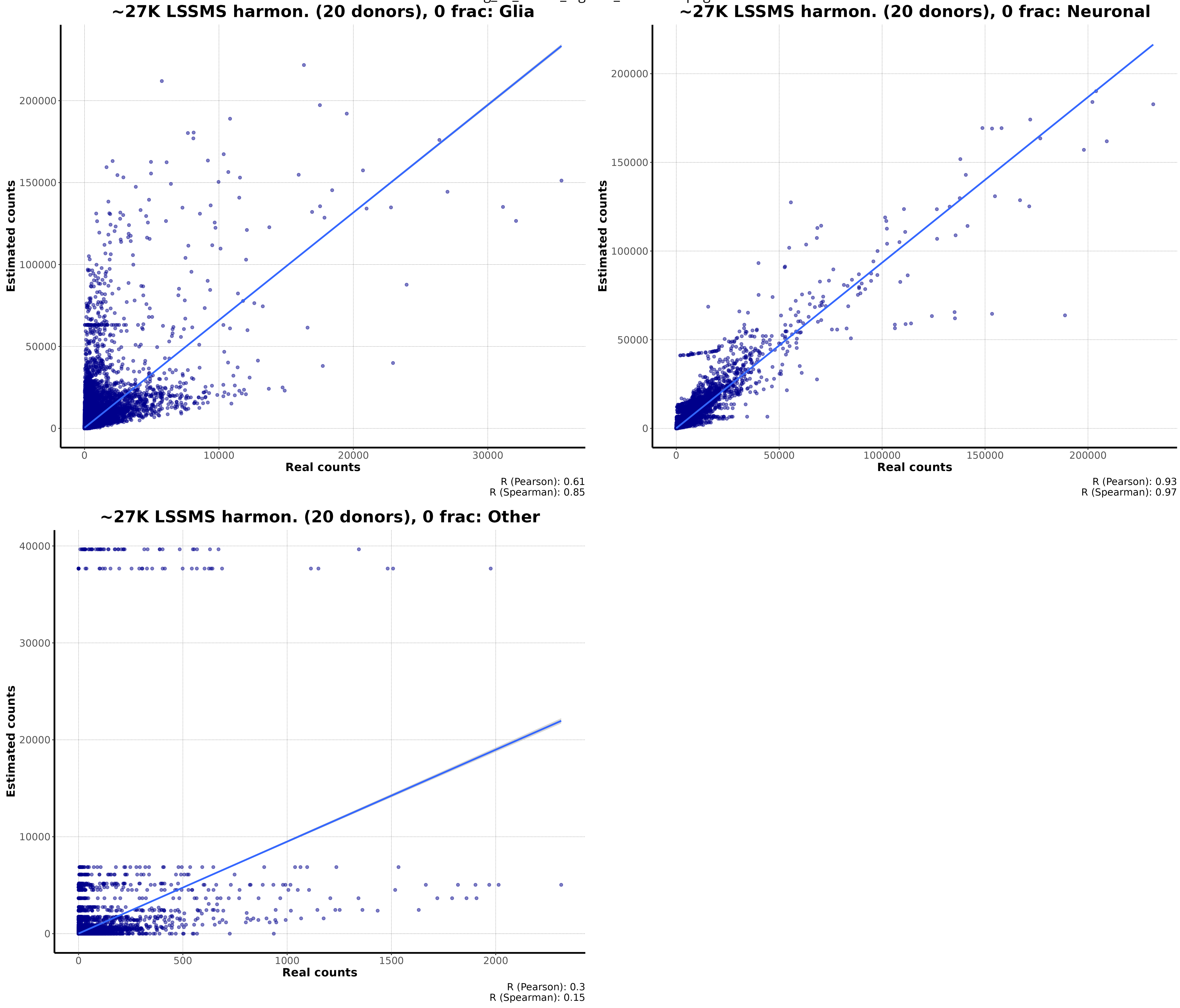

Fig_S9_Sampled_signature_highres_validation.png

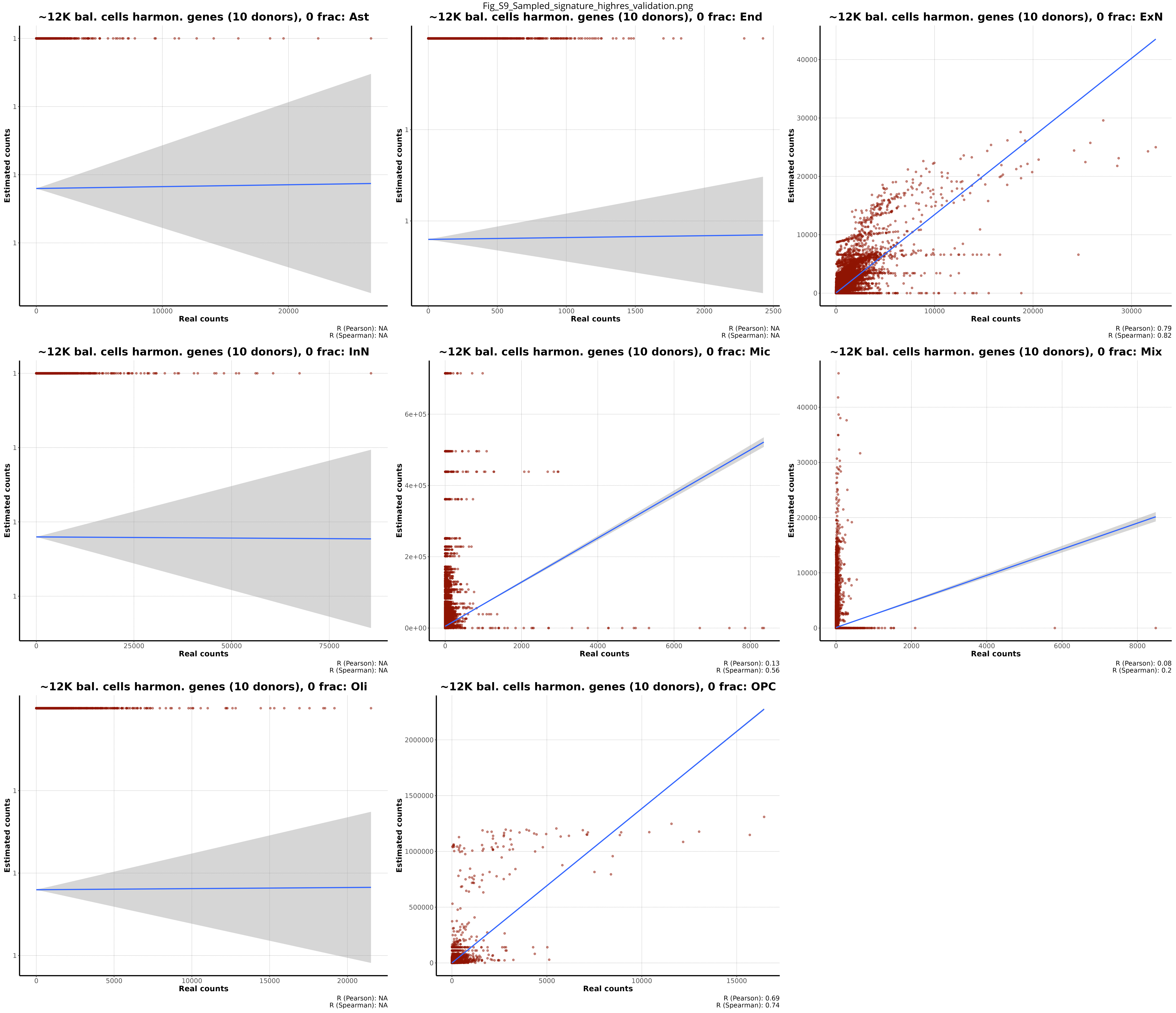

Fig_S10_DLPFC_GO_BP_bar.png

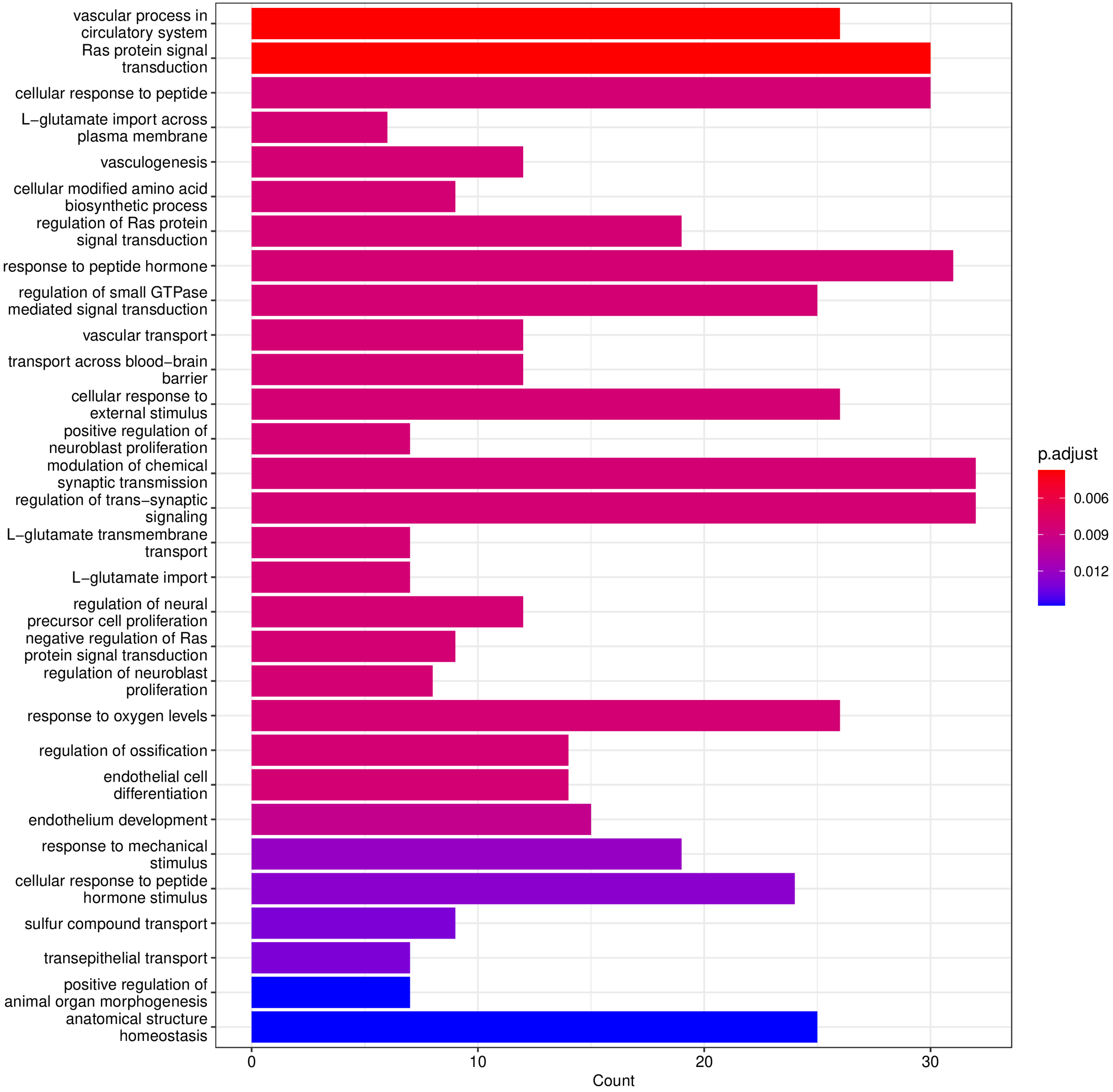

Fig_S11_DLPFC_GO_CC_bar.png

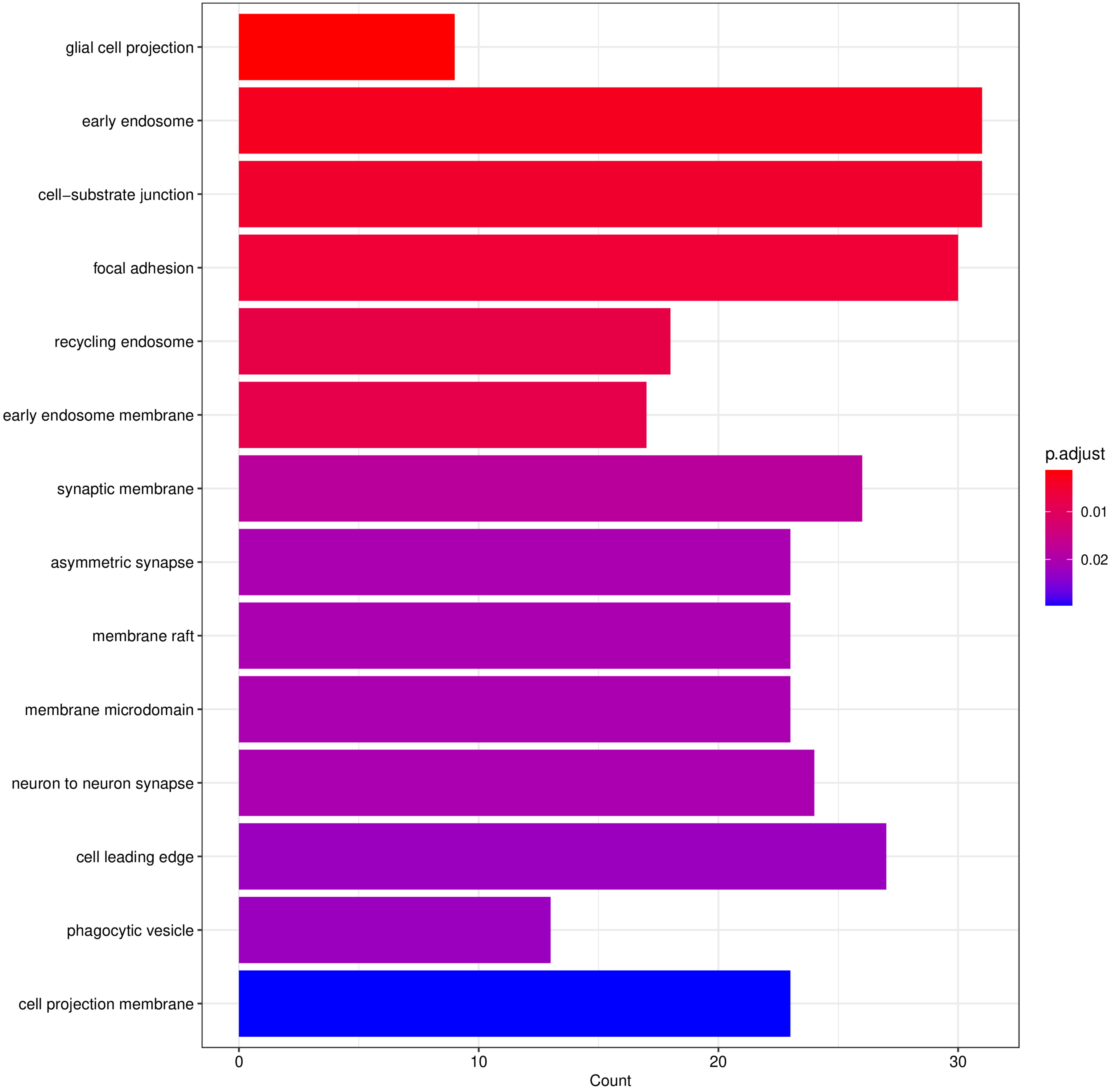

Supplement: Supplementary file 2 — Fig_S1_S11 [file 41398_2026_3978_MOESM2_ESM.pdf]
